# Syntaxin 18 regulates the DNA damage response and epithelial-to-mesenchymal transition to promote radiation resistance of lung cancer

**DOI:** 10.1038/s41419-022-04978-4

**Published:** 2022-06-06

**Authors:** Clotilde Thumser-Henner, Sebastian Oeck, Sophie Kalmbach, Jan Forster, Franziska Kindl, Ali Sak, Alexander Schramm, Martin Schuler

**Affiliations:** 1 Laboratory of Molecular Oncology, Department of Medical Oncology, West German Cancer Center, University Hospital Essen, Essen, Germany; 2 German Cancer Consortium (DKTK), Partner site University Hospital Essen, Essen, Germany; 3 Department of Human Genetics, University Hospital Essen, Essen, Germany; 4 Department of Radiotherapy, University Hospital Essen, Essen, Germany

**Keywords:** Radiotherapy, Non-small-cell lung cancer

## Abstract

Radiotherapy is an important modality in lung cancer treatment. Despite advances in treatment planning and dose delivery, patient benefit is still limited by in-field relapse and metastatic recurrence. Simultaneous application of cisplatinum-based chemotherapy leads to moderately improved outcomes, thus providing proof-of-concept for radiosensitization strategies in lung cancer. In an unbiased functional genetic screen for radiosensitization targets in lung cancer, we identified syntaxin 18, a protein involved in retrograde vesicular transport between the Golgi apparatus and endoplasmic reticulum, as mediator of radioresistance. Downregulation of endogenous syntaxin 18 specifically reduced clonogenic survival of radioresistant and radiosensitive lung cancer cells following X-radiation. Gene expression programs regulating DNA repair, mitotic checkpoints and mitosis were altered in isogenic cells with reduced syntaxin 18 expression. Functionally, this translated into impaired DNA damage-induced cell cycle checkpoints leading to cell death by mitotic catastrophe. Interestingly, downregulation of syntaxin 18 in lung cancer cells also impaired expression of markers of epithelial-mesenchymal-transition, and reduced migration and invasion capacity. These findings suggest that syntaxin 18 is a key player regulating genes responsible for controlling the growth of the primary tumor as well as metastases upon radiotherapy of lung cancer. They provide a promising lead for biologically rational radiosensitization strategies impacting on radiation-induced cell death as well as metastasis.

## Introduction

Lung cancer is the leading cause of cancer-related deaths worldwide and 85% of patients are diagnosed with non-small-cell lung cancer (NSCLC). Of these, 70% are diagnosed with advanced or metastatic disease. Curative treatment options for patients with locally advanced NSCLC (WHO stage III) include radiotherapy, chemotherapy and surgery. While meta-analyses have confirmed that concurrent chemoradiotherapy produces superior survival outcomes [1], the radiosensitization effect of simultaneously administered cisplatinum-based chemotherapy is moderate at best. Genomic alterations of patients are guiding treatment personalization in patients with metastatic NSCLC and in adjuvant treatment of surgically resected EGFR-mutated NSCLC [2]. However, they are not considered for the selection of radiosensitizing agents in chemoradiotherapy of NSCLC. Radioresistance within the primary tumor as well as by metachronous metastases remains a major cause of treatment failure in locally advanced NSCLC [3]. Hence, identifying new targets for personalized radiosensitization strategies remains a priority. To that aim, studies on radioresistance mechanisms have led to the development of new treatment strategies such as the targeting of DNA repair pathways and cell cycle checkpoints, including ataxia-telangiectasia mutated (ATM) and poly (ADP-ribose) polymerase inhibitors [4, 5]. In the light of these findings, we performed an unbiased functional in vitro screen based on a lentiviral shRNA library to identify novel modulators of radiation response in NSCLC. We identified syntaxin 18 (STX18), a protein involved in retrograde vesicular transport between the Golgi apparatus and endoplasmic reticulum [6, 7], as a potential modulator of radiosensitivity. Mechanistic studies in a NSCLC model place STX18 in the regulation of radiation-induced cell cycle checkpoints. Interestingly, STX18 also impacts cellular phenotypes and functionalities involved in the metastatic process. These findings highlight STX18 and its targeted pathways as a new avenue for radiosensitization of patients with lung cancer.

## Results

### An unbiased in vitro screen identifies potential modulators of radiosensitivity

**Fig. 1:**
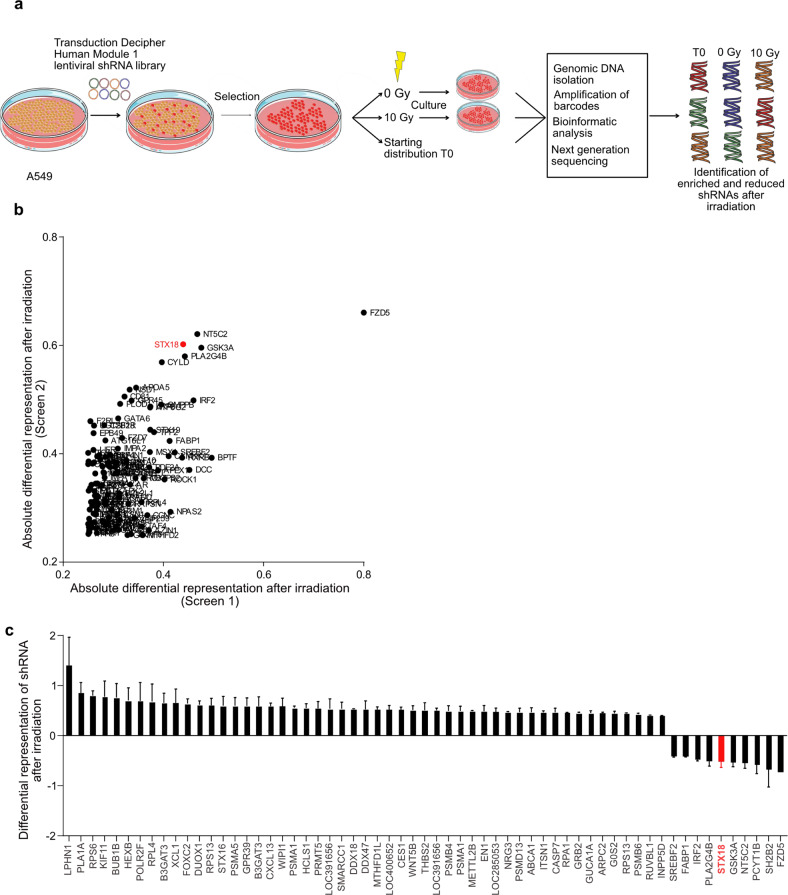
An in vitro shRNA screen identifies STX18 as a modulator of radiosensitivity in A549 cells. **a** A549 cells were transduced with the DECIPHER shRNA library Human Module 1, comprising 27500 pooled shRNA with a transduction efficiency of 30% and cells were selected with puromycin. After 5 days, cells were pooled and either frozen, irradiated with 10 Gy or not irradiated. DNA was then isolated and sequenced. Analysis distinguished barcodes that were enriched or reduced after irradiation. *n* = 2. **b** Differential representation of individual shRNA with highest reduction after irradiation in both repetitions of the screen. Data were normalized to shRNA representation in the initial cell population. **c** Normalized differential expression of the most enriched and reduced shRNA after irradiation. Data were normalized to shRNA representation in the initial cell population.

To identify candidates for modulation of radiosensitivity in NSCLC, we used A549 human lung cancer cells that were lentivirally transduced to express a barcoded-shRNA library targeting more than 5000 genes enriched in signaling pathway targets (Fig. 1a). After X-radiation, sequencing of the barcodes and their comparative analysis in surviving cells identified shRNA that were either enriched or depleted after irradiation when compared to controls, indicating that targeted genes conferred radiosensitivity or radioresistance, respectively (Fig. 1a–c and Supplementary Table 1). As we were most interested in identifying genes that contributed to resistance, we focused on shRNA that were at least 40% less represented in the irradiated samples. Frizzled 5 (FZD5)-targeting shRNA was prominently reduced after irradiation; however, its knockdown did not affect radioresistance of A549 cells in validation experiments (Supplementary Fig. 1). From the other candidate top-ranked genes STX18 was selected for further validation and mechanistic exploration, as this gene had not been described in the context of radiosensitivity before (Fig. 1b, c).

### STX18 knockdown increases radiosensitivity of A549 and H460 cells

**Fig. 2:**
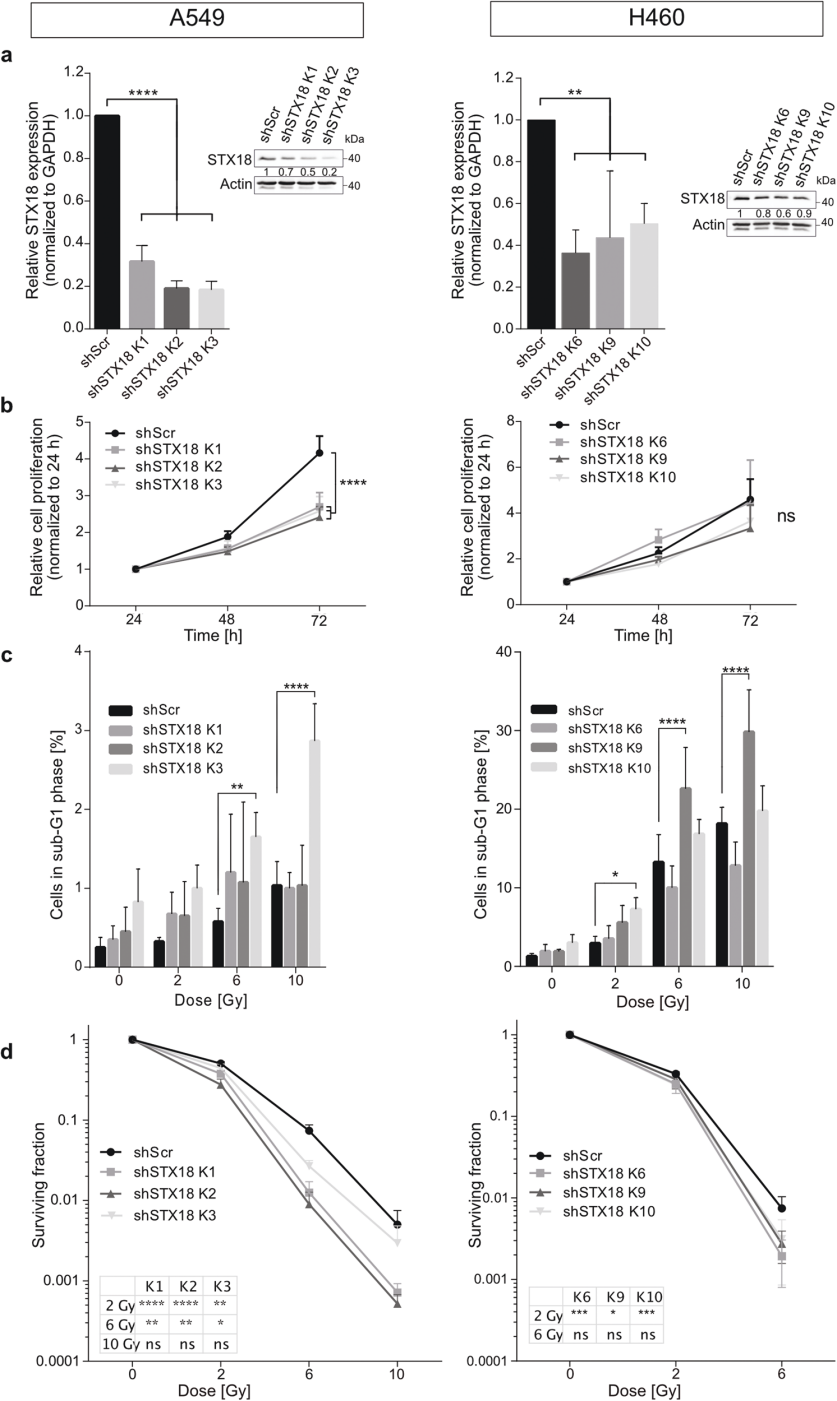
STX18 knockdown sensitizes radioresistant A549 and radiosensitive H460 cell lines to irradiation. **a** A549 and H460 cells were transduced with a shRNA targeting STX18 and its expression was quantified by RT-qPCR. *n* = 3–4. The insert shows respective immunoblotting analyses of STX18 expression. Here, quantification was achieved after normalization to the loading control (Actin) and the shScr sample. *n* = 3. **b** Proliferation of unirradiated cells was measured by proliferation/cell viability assay. *n* = 3. **c** Quantification of sub-G1 fraction by flow cytometry after irradiation. Cells were irradiated and cell cycle distribution was quantified by PI staining after 72 h. *n* = 3–5. **d** Colony formation ability was assessed after irradiation. Cells were irradiated and colonies were counted after 10 days. *n* = 3.

To validate STX18 as a modulator of radiosensitivity, A549 and H460 cells were stably transduced with a shRNA targeting STX18. In three independent single cell clones, STX18 mRNA expression was significantly decreased (by ~80% for A549; by ~50% in H460 cells) (Fig. 2a). Reduced STX18 levels were confirmed by immunoblotting in both cell lines (Fig. 2a). Proliferation of A549 cells, but not H460, was decreased when STX18 was downregulated (Fig. 2b). To study the effect of STX18 knockdown on short-term survival, cells were irradiated, and cell cycle distribution was analyzed 72 h later. Both A549 shSTX18 cells and H460 shSTX18 cells displayed increased sensitivity to irradiation with marked differences between cell clones and a lower apoptosis rate in A549 cells compared to H460 cells (Fig. 2c and Supplementary Fig. 2). STX18 knockdown significantly impaired long-term survival after irradiation as deduced from colony formation capacity in both cell lines (Fig. 2d). Increased apoptosis and impaired survival upon STX18 knockdown were also confirmed using an independent shRNA (Supplementary Fig. 3). Reintroduction of STX18 in A549 shSTX18 cells (Supplementary Fig. 4a, b) led to decreased apoptosis after high-dose irradiation, restoring the sensitivity to the level of the control cell line (Supplementary Fig. 4c). Reintroduction of STX18 also mitigated the radiosensitizing effect mediated by STX18 knockdown on long-term survival (Supplementary Fig. 4d). Interestingly, overexpression of STX18 in A549 parental cells did not alter the response to radiation regarding apoptosis and clonogenic survival (Supplementary Fig. 4e–h).

### STX18 impacts on cell cycle checkpoint arrest and mitotic entry

**Fig. 3:**
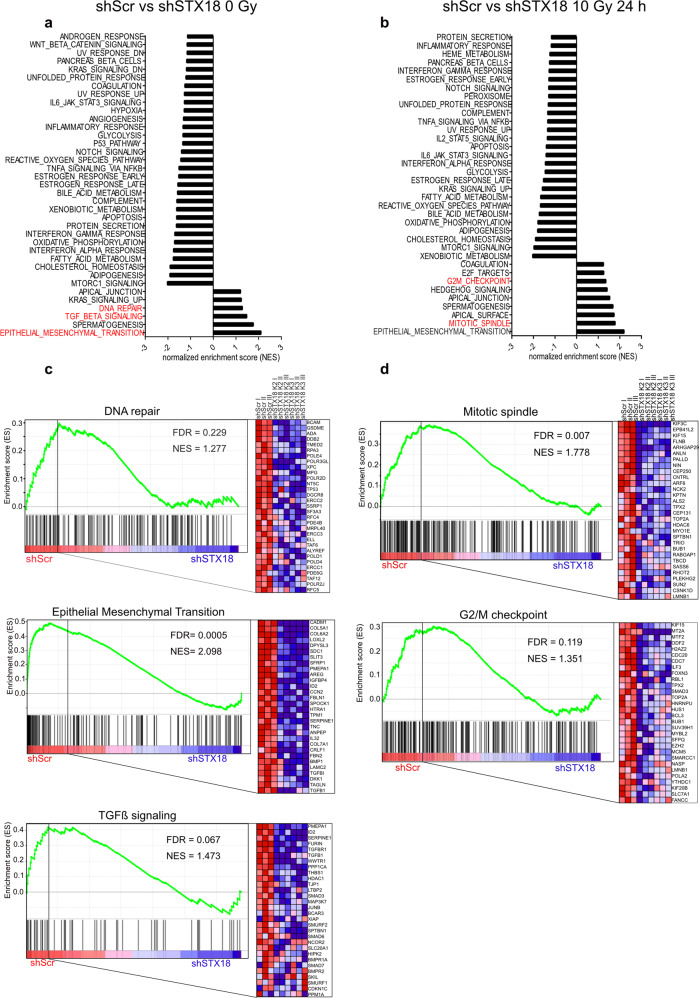
RNA profiling of A549 cells identifies differentially expressed gene sets after STX18 knockdown and irradiation. Differentially represented gene sets in shScr vs shSTX18 at basal level (**a**) and 24 h after irradiation with 10 Gy (**b**). Only the gene sets with an FDR < 0.25 are represented. For representation of A549-shSTX18 cells, counts from A549-shSTX18 K2 and K3 were pooled. **c** Enrichment plots of the gene sets “DNA repair”, “epithelial mesenchymal transition” and “TGFβ signaling” enriched in A549-shScr vs A549-shSTX18 K2 and K3. Biological replicates are indicated with I, II, and III. The 30 most enriched genes for each gene sets are represented. **d** Enrichment plots of the gene sets “mitotic spindle” and “G2/M checkpoint” enriched in A549-shScr vs A549-shSTX18 K2 and K3 after irradiation. Biological replicates are indicated with I, II, and III. The 30 most enriched genes for each gene sets are represented. *n* = 3.

To identify possible targets of STX18, RNA profiling of A549 cells with and without STX18 knockdown was performed before and after irradiation with 10 Gy. Using gene set enrichment analysis (GSEA), we identified genes and pathways differently expressed between the control cell lines and two clonal cell lines. Comparison of A549 cells transfected with a control shRNA (A549-shScr) and A549-shSTX18 (K2 and K3) cells showed a significant enrichment of gene sets including DNA repair, epithelial-mesenchymal transition (EMT) and transforming growth factor β (TGFβ). All these gene sets were more highly expressed in the control cell line (Fig. 3a, c), however, enrichment of “DNA repair” did not reach statistical significance. Interestingly, comparison of A549-shScr and A549-shSTX18 cells highlighted an enrichment of genes involved in the G2/M checkpoint and mitotic spindle checkpoint 24 h following irradiation with 10 Gy (Fig. 3b, d), while the overarching gene set “DNA repair” was not recovered.

**Fig. 4:**
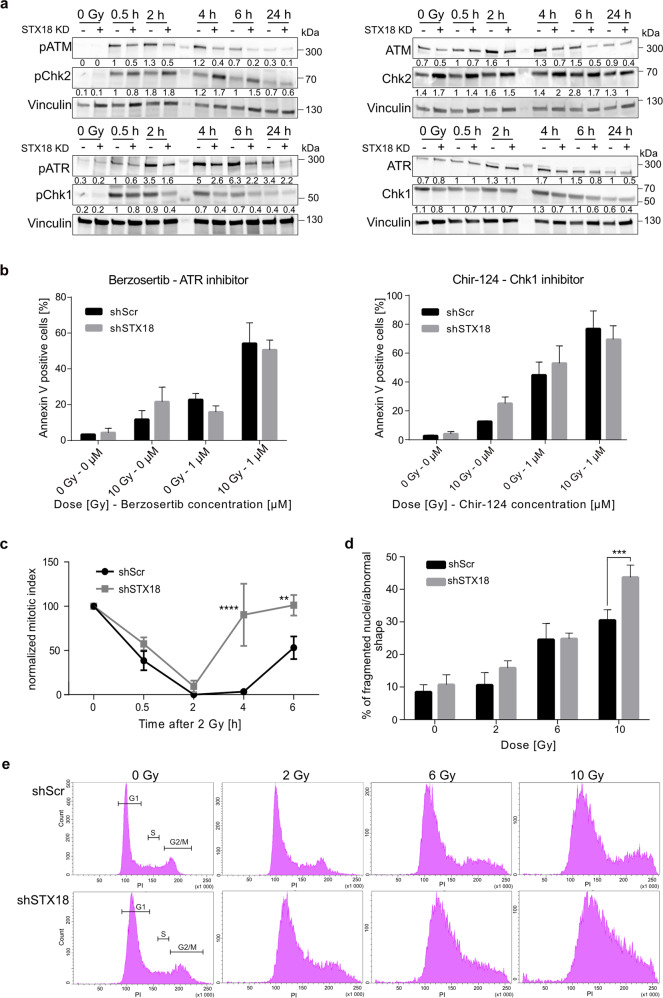
STX18 knockdown leads to a defect in G2/M checkpoint in line with a premature entry into mitosis in A549 cells. For the representation of STX18 knockdown, A549-shSTX18 K3 cells were used. **a** Immunoblotting analysis of proteins involved in cell cycle checkpoints after 10 Gy. Vinculin was used as control. After normalization to the loading control, the samples were compared to the shScr-0.5 h sample. **b** Detection by flow cytometry of Annexin V positive cells following irradiation with 10 Gy and/or pre-treatment with 1 µM berzosertib or Chir-124. Cells were incubated for 72 h then stained with Annexin V. **c** Quantification of mitotic index after irradiation with 2 Gy by quantification of cells positive for phosphorylation of Histone H3 by flow cytometry. **d** Quantification of fragmented nuclei and nuclei with abnormal shape in the cell population 72 h after irradiation. **e** Cell cycle analysis by flow cytometry after irradiation. Cells were irradiated and cell cycle distribution was analyzed by PI staining after 72 h. *n* = 3 for all experiments.

To validate the involvement of STX18 in regulating cell cycle checkpoint associated proteins, expression and activity of ATM and ataxia telangiectasia and rad3 related (ATR) as well as their downstream targets checkpoint kinase (Chk) 2 and 1, respectively, were analyzed in A549 cells after irradiation with 10 Gy (Fig. 4a) while H460 cells, which are more sensitive to irradiation, were subjected to 6 Gy (Supplementary Fig. 5a). Levels of total and phosphorylated forms of ATM were decreased after irradiation in both cell lines upon STX18 knockdown. However, neither total Chk2 levels nor its phosphorylation were markedly affected by STX18 knockdown. While levels of total ATR were comparable between the control samples and cells with STX18 knockdown in A549 cells, ATR phosphorylation was reduced in A549-shSTX18 cells after irradiation. H460 cells showed a reduction in total ATR and its phosphorylation as a consequence of STX18 knockdown. Irradiation-induced activation of Chk1, as assessed by probing for the phosphorylated form, was less pronounced when STX18 was downregulated in A549 but increased in the H460 set. Furthermore, the expression of the p53 tumor suppressor protein, involved in G1/S and G2/M checkpoints, was decreased in A549-shSTX18 cells at baseline as well as following X-radiation (Supplementary Fig. 6a). To functionally validate these results, apoptosis was quantified after treatment with irradiation, berzosertib, an ATR inhibitor, and Chir-124, a Chk1 inhibitor. As expected, STX18 knockdown in A549 (Fig. 4b) and H460 (Supplementary Fig. 5b) cells resulted in significantly higher radiosensitivity. Treatment with berzosertib attenuated radioresistance of shScr cells to the level of irradiation-induced cell death of shSTX18 cells. Furthermore, pre-treatment with berzosertib or Chir-124 sensitized A549-shScr to the same extent as A549-shSTX18 cells. Here, H460 cells showed similar effects but were intrinsically more sensitive to both inhibitors. Additionally, downregulation of STX18 in H460 cells was associated with increased dependency on Chk1 signaling after irradiation (Supplementary Fig. 5a) as indicated by higher sensitivity to Chk1 inhibition. To analyze possible defects in the G2/M checkpoint, phosphorylation of Histone H3 at Ser-10 (H3pS10, a mitotic marker) was quantified by flow cytometry after irradiation in A549 cells. The fraction of cells in mitosis decreased was almost undetectable regardless of the STX18 expression, in line with an intact G2-checkpoint upon irradiation (Fig. 4c). However, the mitotic index of A549-shSTX18 cells started increasing after four hours, while control cells had a prolonged G2 checkpoint (Fig. 4c). To investigate if early re-entry into mitosis caused by STX18 knockdown also affected DNA repair, γH2A.X foci were quantified after irradiation. The number of foci per cell was significantly reduced after 30 min in H460-shSTX18 cells (Supplementary Fig. 5e) and after 30 min and 2 h in A549-shSTX18 cells (Supplementary Fig. 6c), while DNA repair kinetics were not impacted by STX18 knockdown (Supplementary Fig. 6d). The reduced number of foci could be attributed to reduced phosphorylation of H2A.X caused by decreased activity of ATR and ATM. STX18 knockdown also had no significant effect on DNA repair efficiency on cellular level (Supplementary Fig. 6e, f). To investigate if knockdown of STX18 cells was associated with mitotic catastrophe, fragmented nuclei and nuclei displaying an abnormal shape were quantified after irradiation. Indeed, the fraction of cells showing nuclear fragmentation was significantly increased in STX18 knockdown cells 72 h after irradiation (Fig. 4d), which can be indicative of cell death following a premature entry into mitosis. In line with these findings, analysis of cell cycle 72 h after irradiation showed an irregular distribution between cell cycle phases in A549- and H460-shSTX18 cells at any dose, while an ordered distribution between the cell cycle phases was maintained in the control cell lines (Fig. 4e and Supplementary Fig. 5c). Specifically, STX18 knockdown resulted in a significantly decreased G2 arrest in H460 (Supplementary Fig. 5d) and A549 (Supplementary Fig. 6b) compared to control cells after irradiation. These findings indicate that STX18 expression correlates with sensing and signaling of DNA damage that impacts on cell cycle regulation upon irradiation.

### STX18 expression affects expression of epithelial-to-mesenchymal transition and migration/invasion capacities

**Fig. 5:**
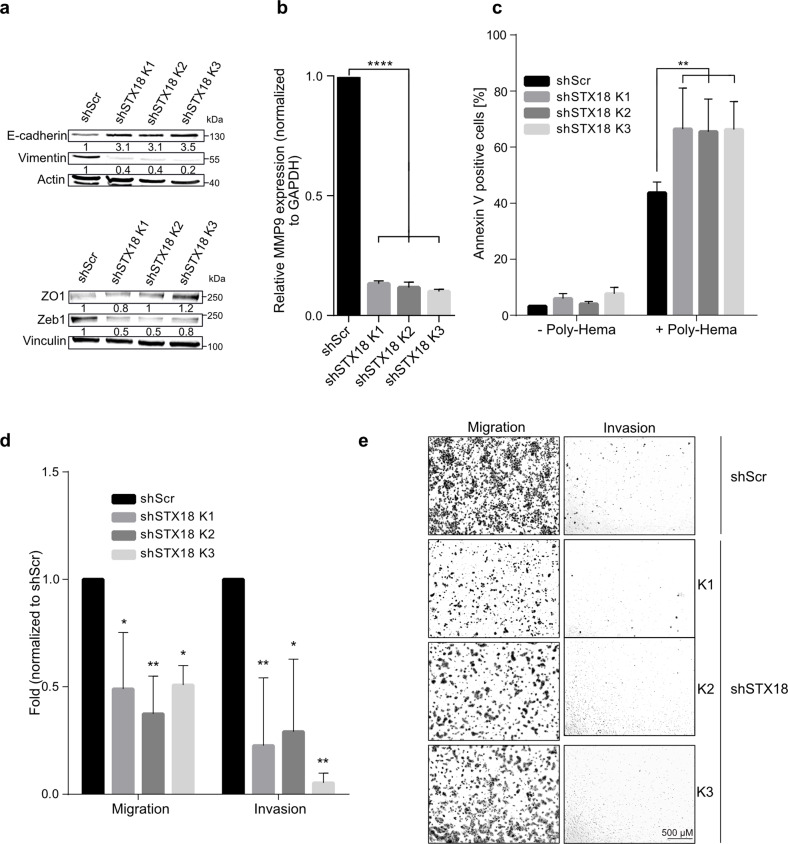
Syntaxin 18 knockdown leads to an epithelial phenotype and decreased migration and invasion capacities in A549 cells. **a** Immunoblotting analysis of E-cadherin, vimentin, ZO-1 and Zeb1 expression. Vinculin and actin were used as controls. After normalization to the loading control, the samples were compared to the shScr sample (set to 1). **b** RT-qPCR analysis of MMP9 mRNA expression. **c** Detection by flow cytometry of Annexin V positive cells. Cells were incubated for 72 h on poly(2-hydroxyethyl methacrylate)-coated (Poly-Hema) plates and stained with Annexin V. **d** Boyden chamber assay for A549 migration and invasion. Cells were seeded on the transwell plate after overnight serum starvation and incubated for 24 h. Migrated and invaded cells were then stained with crystal violet. **e** Representative pictures from Boyden chamber assay results shown in (**d**). Scale bar, 500 µm. *n* = 3 for all experiments.

As indicated above, RNA profiling of A549 cells with high or low levels of STX18 and subsequent GSEA highlighted an enrichment of genes involved in EMT and TGFβ signaling in the former (Fig. 3a, c). To validate if expression of EMT markers were directly affected by STX18 knockdown, their protein expression was assessed by immunoblotting. A549-shSTX18 cells showed an increased expression of the epithelial status marker E-cadherin, while the expression of the mesenchymal markers vimentin and zinc finger e-box binding homeobox 1 (Zeb1) was decreased in three independent clones (Fig. 5a). Furthermore, expression of the matrix metalloproteinase (MMP) 9 gene was strongly impaired after STX18 knockdown (Fig. 5b). As mesenchymal phenotypes are often associated with anchorage-independent growth, the effect of STX18 expression levels on anoikis sensitivity was assessed. To this end, A549 cells were plated on wells coated with poly(2-hydroxyethyl methacrylate), mimicking the detachment of the cells from the matrix. After 72 h, the fraction of cells undergoing apoptosis was significantly higher in A549-shSTX18 cells (Fig. 5c). Additionally, both migration and invasion capacities were significantly decreased in STX18 downregulated cells (Fig. 5d, e). These results support the hypothesis that STX18 plays a role in the regulation of EMT and metastatic capacities of A549 cells.

## Discussion

Radiotherapy represents a mainstay for treating NSCLC. In the last decades, significant technological progress in stereotactic body radiation therapy has led to improved targeting of the tumor, while sparing the surrounding healthy tissue. Thus, radiotherapy is now widely used for the ablation of NSCLC primary tumors in patients who are not suitable for surgery [8, 9]. In locally advanced NSCLC, the efficacy and curative potential of radiotherapy are enhanced by concurrent administration of cisplatin-based chemotherapy [1]. However, the effect of this non-specific radiosensitization approach is rather modest. While the discovery of actionable driver mutations led to the development of personalized targeted molecular therapy, chemoradiotherapy is by and large still in its infancy with respect to accounting for inter-patient heterogeneity [10]. Although radioresistance is one of the main factors underlying local recurrence, chemoradiotherapy currently does not consider tumor heterogeneity. Among others, the PACIFIC trial’s concurrent treatment with the anti-PD-L1 antibody durvalumab to curatively intended chemoradiotherapy in stage III NSCLC patients provided proof-of-concept for improvement of survival outcomes by molecularly targeted pharmacological interventions in conjunction with radiotherapy [11–13]. Against this background, a recent study identified an association between KEAP1/NFE2L2 mutations and local failure of NSCLC. Furthermore, these mutations were overrepresented in patients who developed radioresistance. Interestingly, radioresistance of KEAP1/NFE2L2 mutated NSCLC cells could be overcome by treatment with a glutaminase inhibitor resulting in depletion of glutathione [14]. Still, the identification of patients who will benefit from radiotherapy alone or in combination with other treatment modalities is lagging. To identify new targets governing the sensitivity of lung cancer cells to irradiation, we performed a functional in vitro screen based on a lentiviral shRNA library and validated the results using two cell lines with KEAP1 mutations and wt-TP53, but different intrinsic radiosensitivity. We identified STX18 as a target modulating cell survival after irradiation. STX18 encodes syntaxin 18, which belongs to the soluble N-ethylmaleimide-sensitive-factor attachment protein receptor family and is involved in retrograde transport from Golgi to the endoplasmic reticulum as well as in endoplasmic reticulum membrane fusion [6, 7]. While the role of STX18 in modulating sensitivity to irradiation is unknown, there are few functional studies suggesting a role for STX18 in cancer cells and in genomic stability. Bassett et al. demonstrated that STX18 knockdown led to increased growth of breast cancer cells in vitro despite its overexpression in breast cancer samples [15]. In a different context, STX18 was described to interact with RINT1 and RAD50 in neuronal cells, thus linking STX18 to genomic stability [16]. Here, we demonstrate that knockdown of endogenous STX18 significantly reduced clonogenic survival of A549 and H460 lung cancer cells following X-radiation. This is, to our knowledge, the first study to show a relationship between a member of the syntaxin family and radiation response.

It is well defined that after DNA damage, ATM/Chk2 and ATR/Chk1 stabilize p53 by phosphorylation, increasing its activity as transcriptional regulator and leading to cell cycle arrest in the G1 phase. This cell cycle checkpoint is absent in cells lacking p53 function [17, 18]. Several reports indicate that defects in this G1/S checkpoint induce a strong dependency on the G2/M checkpoint to preserve genomic integrity. The latter is governed by ATR/Chk1 expression and activity serving to avoid premature mitotic entry that eventually leads to cell death due to mitotic catastrophe or other nuclear abnormalities. Therefore, inhibition of the ATR/Chk1 axis is thought to specifically sensitize p53-deficient or p53-mutated cells [19–22]. These results suggest a fundamental role of Chk1 in G2/M checkpoint regulation. We found STX18 knockdown to be associated with decreased ATR/Chk1 activity after irradiation, as well as a decreased irradiation-induced stabilization of the p53 protein in A549. Furthermore, STX18 knockdown correlated with a decreased fraction of cells in the G2-phase of the cell cycle and increased irradiation-induced nuclear fragmentation in these cells, all of which support the idea of an STX18-mediated regulation of entry into mitosis after irradiation in A549 cells. However, in line with the findings of Tao, Leteur et al., this was not associated with an apparent defect in DNA damage repair [22]. The molecular scaffold to explain STX18-mediated effects is largely unknown, however, it was recently suggested that SNAP29 forms a complex together with STX18 and Sec22b at the ER and the Golgi apparatus. While we could not confirm increased co-localization of SNAP29 with STX18 upon irradiation in our cell lines models [23], Morelli and colleagues have demonstrated spindle alterations and increased formation of micronuclei in neuroepithelial stem cells after SNAP29 depletion, indicating the involvement of this protein complex in mitosis [24]. Although the underlying mechanisms remain to be explored, we hypothesize that knockdown of STX18 affects p53 stability after irradiation, rendering A549 cells addicted to the G2/M checkpoint to avoid premature mitotic entry. Since ATR/Chk1 signaling is impaired, reduced function of both cell cycle checkpoints provokes an increase in the fraction of cells entering mitotic catastrophe and subsequent cell death. It would be interesting to analyze the role of STX18 in p53-mutated cells as well in other genetic conditions frequently found in non-small-cell cancer (KRAS mutations, aberrant receptor tyrosine kinase activation) to delineate if the effects of STX18 are restricted to wt-TP53 tumors. Nevertheless, our manuscript provides clues and evidence that STX18 is an important player in the radiation response in non-small-cell lung cancer, while genetic features modulating dependency on STX18 remain to be identified in future work. In addition, it remains to be determined how the cell-autonomous effects described here will transfer to a situation in which tumor cells are in contact with other cell types, e.g., in an in vivo model.

In addition to linking STX18 and the radiation response via major determinants of cell cycle regulation, RNA profiling of A549 cells also revealed that STX18 levels affect TGFβ-induced pathways and EMT. We showed that STX18 expression promoted a mesenchymal phenotype, with elevated expression of mesenchymal-associated markers in addition to increased migration and invasion abilities. This is in line with findings of others demonstrating the involvement of syntaxin 1 in glioblastoma cell invasion, while expression of syntaxin 6 expression was associated with increased migration of esophageal cancer cell [25, 26]. It remains to be investigated if the role of STX18 in radioresistance and metastatic abilities are connected or represents two distinct functionalities of STX18 acting in separate pathways.

Altogether, our findings highlight a new link between vesicular transport, radioresistance and metastatic progression, which constitute two hallmarks of failure of radiotherapy in locally advanced NSCLC. The effects observed from cells with different levels of STX18 might hint at novel strategy to exploit cell cycle checkpoint deficiency for enhancing the efficacy of radiotherapy in NSCLC and simultaneously suppressing its metastatic potential.

## Materials and methods

### Cell culture

All cell lines were purchased from ATCC (Manassas, VA, USA) and authenticated by STR analysis. The absence of mycoplasma was tested regularly by PCR. A549 and NCI-H460 cells were cultured in RPMI 1640 (Gibco, Waltham, MA, USA); HEK293FT and Phoenix-Eco cells were cultured in DMEM (Gibco). All cell media were supplemented with 10% FBS (Biochrom, Cambridge, UK). All centrifuging steps were performed at 300 × *g* for 5 min if not stated otherwise.

### Irradiation

Irradiation was performed using a RS320 X-ray irradiator (X-Strahl Life Sciences, Camberley, UK) kindly provided by the working group of Prof. Stuschke (Dept. of Radiation Oncology, University Hospital Essen). Cells were irradiated at 300 kV and 10 mA.

### Transfection and lenti/retro-viral transduction

FuGENE (Promega, Madison, WI, USA) was used to transfect cells at a ratio of 2:1 (ratio FuGENE to DNA), according to the manufacturer’s protocol. Lentiviral and retroviral particles were generated in HEK293FT and Phoenix-Eco cells, respectively. For transduction, 30,000 cells were seeded in six-well plates and incubated for 24 h with viral particles supplemented with 1 µg/mL polybrene (Sigma, St. Louis, MO, USA) to enhance transduction efficiency. Cells were then selected with 1 µg/mL puromycin (Calbiochem, San Diego, CA, USA), or 800 µg/mL neomycin (Thermo Fisher Scientific, Waltham, MA, USA) for 7 days. Single cell clones were generated using limiting dilution. Lentiviral STX18 shRNA and scrambled controls were obtained from Sigma. For reintroduction of STX18, an empty vector (Mscv.PG.Neo) and a plasmid carrying STX18 (Mscv.STX18) were used. For sequences of shRNA, see Supplementary Table 2.

### In vitro screen and identification of genes involved in radioresistance

The DECIPHER shRNA Library Human module 1 (Cellecta, Mountain View, CA, USA), consisting of 27,500 pooled shRNA targeting 5043 signaling pathway genes was used to perform a screen to compare the shRNA distribution prior to and after irradiation of A549 cells. Briefly, HEK293FT were transfected with the shRNA library as described previously [27]. Viral particles were collected and filtered 72 h after transfection. A549 cells were transduced with an amount of viral particles ensuring an efficiency of 30%. Cells were then selected with 1 µg/mL puromycin for 5 days and divided into three samples: one sample was frozen, one was irradiated with 10 Gy and the last one was not irradiated. Three days after irradiation, DNA was isolated (Qiagen Genomic-tip 500/G, according to the manufacturer’s protocol, Hilden, Germany) and barcoded shRNA were sequenced (BioCat, Heidelberg, Germany).

### RNA isolation and RT-qPCR

RNA was isolated using High Pure RNA isolation Kit (Roche) and 1 µg of RNA was reverse transcribed (Transcriptor High Fidelity cDNA Synthesis Kit, Roche) according to the manufacturer’s protocol. Gene expression was quantified by using SYBR Green as a fluorescent probe and primers specified in Supplementary Table 3.

### RNA sequencing

RNA was isolated from cells using RNeasy Mini kit (Qiagen) according to the manufacturer’s protocol. 3’UTR mRNA-Seq library preparation and analysis were performed on an Illumina HiSeq2500. Transcript level quantification was performed using salmon v.0.14.1 [28] against an index built on the ENSEMBL GRCh38 reference transcriptome (release version 100). Transcript level expression was later transferred to gene level by summing all transcript counts per gene. Gene counts from RNA-seq were used for GSEA using the GSEA software (Broad Institute, Cambridge, MA, USA) and the curated Hallmarks gene set [29, 30]. Parameters were set to 1000 permutations and gene set permutation mode. Only enriched gene sets showing a false discovery rate (FDR) below 0.25 were considered.

### Protein isolation and immunoblotting

Proteins were isolated using a 1% NP40 (Fluka, Buchs, Switzerland) buffer containing cOmplete protease inhibitor cocktail (4%, Roche, Basel, Switzerland) and phosphatase inhibitors cocktails 2 and 3 (1% each, Sigma). 30 µg of proteins were loaded on a 4–15% gradient gel (Bio-Rad, Hercules, CA, USA) and subsequently transferred onto a nitrocellulose membrane. Membranes were blocked, and antibodies diluted in 5% BSA (Bovine Serum Albumin, Roth) Tris-buffered saline with Tween-20. If not stated otherwise, all antibodies were diluted 1:1000. The following antibodies were used for immunoblotting: beta-Actin (MP Biomedicals, Santa Ana, CA, USA, #691002), STX18, p53 (Santa Cruz Biotechnology, Dallas, TX, USA, #293067, #71817, respectively), Frizzled 5, E-cadherin, ZO-1, Vimentin, Zeb1 (all from Cell Signaling Technologies, #5266, #3195, #5406, #5741, #3396), Vinculin (1:2000, Abcam, #129002), phospho-ATR, ATR, phospho-Chk1, Chk1, phospho-ATM, ATM, phospho-Chk2, Chk2 (all from Cell Signaling Technologies, #58014, #13934, #2348, #2360, #13050, #2873, #2669, #2662), ATR (Cell Signaling Technologies, #13934). Secondary antibodies were HRP-conjugated (Pierce Antibodies, 1:2500). Western Blot densitometry was performed using the Fiji software (https://fiji.sc/ (Accessed on 20. January 2022)). Bands were normalized to respective loading controls and show the mean of three independent experiments. Means, standard deviation (SD) and statistical analysis are included in Supplementary Table 4.

### Proliferation assay

Proliferation of cells was assessed using 3-(4,5-Dimethylthiazole-2-yl)-2,5-diphenyl-2H- tetrazolium bromide (MTT, Roth, Karlsruhe, Germany). 2000–4000 cells were seeded in a 96-well plate. After the indicated timepoint, 0.5 mg/mL MTT was added to the wells, and 4 h later, solubilization solution was added (10% SDS, 0.01 M hydrochloric acid) and the plate was incubated overnight at 37 °C. The absorbance was then measured at 595 nm in a plate reader (iMark, Bio-Rad).

### Cell cycle analysis

To analyze the effect of irradiation on the cell cycle distribution, 40,000 cells were seeded in six-well plates. After adherence, cells were irradiated and 72 h later, cells were collected and incubated with Propidium/Iodide (PI) in the dark at 4 °C for at least 30 min. Cells were analyzed using a FACS Celesta (BD BioSciences, Franklin Lakes, NJ, USA).

### Apoptosis quantification by Annexin V staining

For determining levels of apoptosis upon irradiation, 40000 cells were seeded in six-well plates and collected 72 h after irradiation. For analysis after treatment, cells were pre-treated with 0.25 or 1 µM Berzosertib (S7102, Selleckchem, Houston, TX, USA) or 0.25 or 1 µM Chir-124 (S2683, Selleckchem) for 2 h before irradiation with 6 or 10 Gy, and collected after 72 h. Staining was accomplished using Annexin V/PI (FITC Annexin V Apoptosis Detection Kit, BD Biosciences) according to the manufacturer’s protocol. Cells were analyzed using a FACS Celesta (BD BioSciences).

For assessment of anoikis, plates were first coated with poly(2-hydroxyethyl methacrylate) (poly-hema, Roth) and incubated overnight at room temperature. Seventy-two hours after seeding, cells were stained and measured as described.

### Colony formation assay

To determine survival upon irradiation, 150–10,000 cells were seeded in six-well plates. Adherent cells were irradiated with doses as indicated. After 10 days, colonies were fixed with 70% ethanol and stained with Coomassie brilliant blue. Colonies formed by more than 50 cells were counted. Plating efficiency and surviving fraction were calculated as follows: plating efficiency= number of colonies formed/number of cells seeded (untreated cells); surviving fraction = (number of colonies formed)/(number of cells seeded) × plating efficiency.

### Quantification of the mitotic index by H3PS10 staining

Cells were harvested after irradiation and fixed in 70% ethanol at −20 °C overnight. After fixation, cells were resuspended in 0.25% Triton X100 in phosphate-buffered saline-Tween (PBS-T), prior to blocking in 1% BSA in PBS-T. Cells were then incubated 90 min with the H3pS10 antibody (Abcam, 1 5000, 1% BSA in PBS-T ab5176). After incubation with the secondary antibody coupled to Alexa Fluor 488 (Thermo Fisher Scientific, 1:300, A21206, in 1% BSA in PBS-T for 1 h), PI (4 µg/ml) and RNAse (0.60 µg/mL) in PBS were added. Samples were analyzed with a flow cytometer (FACS Celesta, BD BioSciences) and the fraction of mitotic cells was determined by normalizing the number of H3pS10 and PI-positive cells to the total number of cells.

### Quantification of nuclear fragmentation

To identify fragmented nuclei upon irradiation, 90,000 cells were seeded and irradiated the next day. Seventy hours later, cells were treated with 0.1 µg/mL colcemid (Gibco) for 2 h. Cells were then collected, centrifuged, and cells were incubated 5 min in 5 mL hypotonic solution (75 mM potassium chloride). After centrifugation, cells were resuspended in fixative solution (methanol and acetic acid mixed 3:1) and incubated overnight at 4 °C. After centrifugation (7 min), cells were washed twice in fixative solution, and resuspended in 2 drops of fixative solution. Samples were spread on each slide and left to dry overnight at 4 °C. Slides were then stained with Giemsa (Roth) (3% in Sorenson buffer, Thermo Fisher Scientific) for 12 min, and rinsed with water. Picture recording and subsequent quantification were achieved using the Axio Scan.Z1 microscope (Zeiss, Oberkochen, Germany, kindly provided by the working group of Prof. Dr. Siveke). Abnormal nuclei were defined by fragmentation, aggregation, or formation of micronuclei. At least 1000 cells per sample were evaluated.

### DNA strand break quantification by γH2A.X staining

To quantify DNA damage upon irradiation, 25,000 cells were seeded on 8-chamber slides (Merck, Darmstadt, Germany). After fixation and permeabilization with 3% PFA, 0.5% Triton X-100 and 8% Sucrose in PBS, cells were blocked with 5% FBS, 5% NGS, 0.2% Triton X-100 in PBS. Next, samples were incubated with the γH2A.X antibody (1:400, Merck, 05-036) for 1 h at room temperature followed by overnight at 4 °C. After washing and incubation with the secondary antibody (anti-mouse Alexa Fluor Plus 488, 1:400, Thermo Fisher Scientific, #A32723), cells were stained with a DAPI/Hoechst33342 mix (1 µg/mL DAPI, 5 µg/mL Hoechst33342 mixed 1:1). Pictures were recorded using an Axio observer fluorescence microscope (Zeiss, kindly provided by the working group of Prof. Dr. Jendrossek, University Hospital Essen) and analyzed with the Focinator 2.0 software, which corrects for the area of the nucleus and allows for quick and semi-automated counting of γH2A.X in at least 50 cells [31].

### Comet assay

130,000 cells were seeded in a six-well plate the day before treatment. The following day, cells were treated with 5 Gy. After the desired timepoint, cells were collected and 1 200 cells were spread on the slide with low-melting point agarose (CometAssay Trevigen, Gaithersburg, MD, USA). Slides were processed according to the manufacturer’s protocol. After drying, slides were stained in SYBR Green (Thermo Fisher Scientific) solution 1:5000 in water for 30 min at room temperature in the dark. Pictures were taken with an EVOS M5000 microscope (Thermo Fisher Scientific) and comet tail lengths were analyzed using ImageJ 1.51j8 [32]. Comet tail length was normalized to the average nuclear diameter of each cell line. At least 50 cells were analyzed.

### Migration/invasion assay

Migration/invasion assay was performed using the BioCoat© GFR Matrigel (Corning, Bedford, MA, USA) plates. After overnight starvation in a medium containing reduced serum levels (0.5%), 40,000 cells were seeded in starvation medium on top of the inserts, which were placed in a 24-well plate filled with 10% FBS medium. After 24 h, cells that did not migrate or invade were removed, and migrated/invaded cells were fixed with 70% ethanol and stained with crystal violet (Roth, Karlsruhe, Germany). Pictures of migrating and invading cells were recorded using a Primovert microscope (Zeiss, Oberkochen, Germany).

### Statistical analysis

On graphs, means ± SD are shown. Significance was estimated for experiments biologically repeated at least three times using GraphPad Prism 6. For comparison of groups involving one variable, one-way ANOVA was used with post hoc test. For comparison of groups involving two variables, two-way ANOVA was used. Both tests were used with Dunnett, Tukey or Sidak post-hoc tests, when applicable. If not stated differently, results can be considered not significant *p* > 0.05. When significant results are shown, *p*-values are indicated as following: *p ≤ 0.05, **p ≤ 0.01, ***p ≤ 0.001, ****p ≤ 0.0001, ns no statistical significance.

## Supplementary information

Supplementary Figures

Supplementary Table 1

Supplementary Table 2

Supplementary Table 3

Supplementary Table 4

Uncropped Western Blots

Dataset 1

Author Checklist

Author contributions

## Data Availability

RNA-sequencing data generated in this study have been deposited in European Nucleotide Archive with the primary accession code PRJEB47277. Full and uncropped western blots are provided as ‘Supplemental Material’.
