# Supplementary material for: Syntaxin 18 regulates the DNA damage response and epithelial-to-mesenchymal transition to promote radiation resistance of lung cancer
Source: Cell Death Dis. 2022 Jun 6;13(6):529. doi: 10.1038/s41419-022-04978-4 (PMC9170725; doi:10.1038/s41419-022-04978-4)
Supplement: Supplementary file 1 — Supplementary Figures [file 41419_2022_4978_MOESM1_ESM.docx]

**Supplementary Figures**


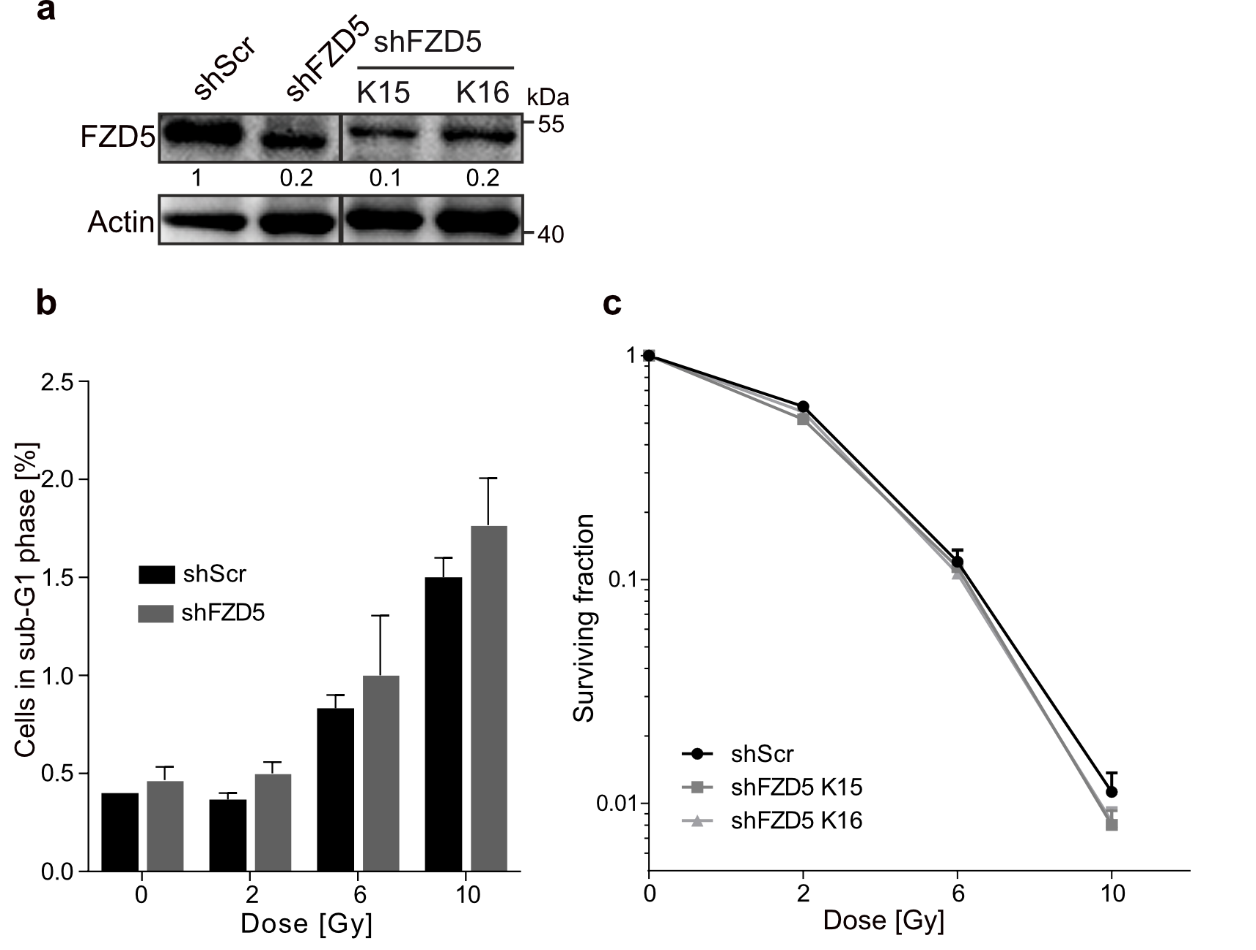


**Supplementary figure 1. Knockdown of FZD5 does not sensitize A549 cells to irradiation.**

**a.** Immunoblotting analysis of FZD5 expression. Actin was used as control. After normalization to the loading control, the samples were compared to the shScr sample (set to 1). n=1. **b.** Quantification of sub-G1 fraction by flow cytometry after irradiation. Cells were irradiated and cell cycle distribution was quantified by PI staining after 72 h. n=3. **c.** Colony formation ability was assessed after irradiation. Cells were irradiated, and colonies were counted after 10 days. n=3.


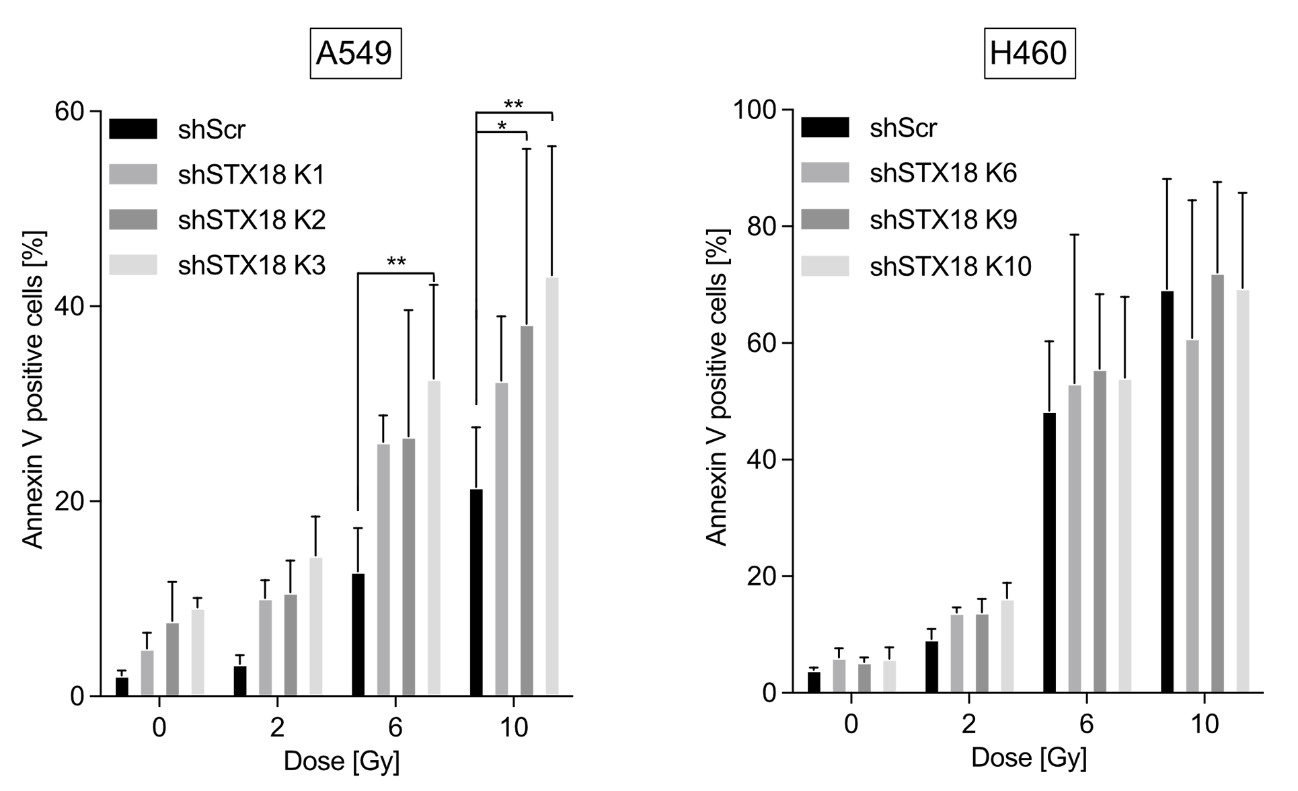


**Supplementary figure 2. Knockdown of STX18 leads to increased apoptosis after irradiation in A549 cells.**

Detection by flow cytometry of Annexin V positive cells following irradiation. A549 and H460 cells were incubated for 72 h after irradiation and stained with Annexin V. n=3.


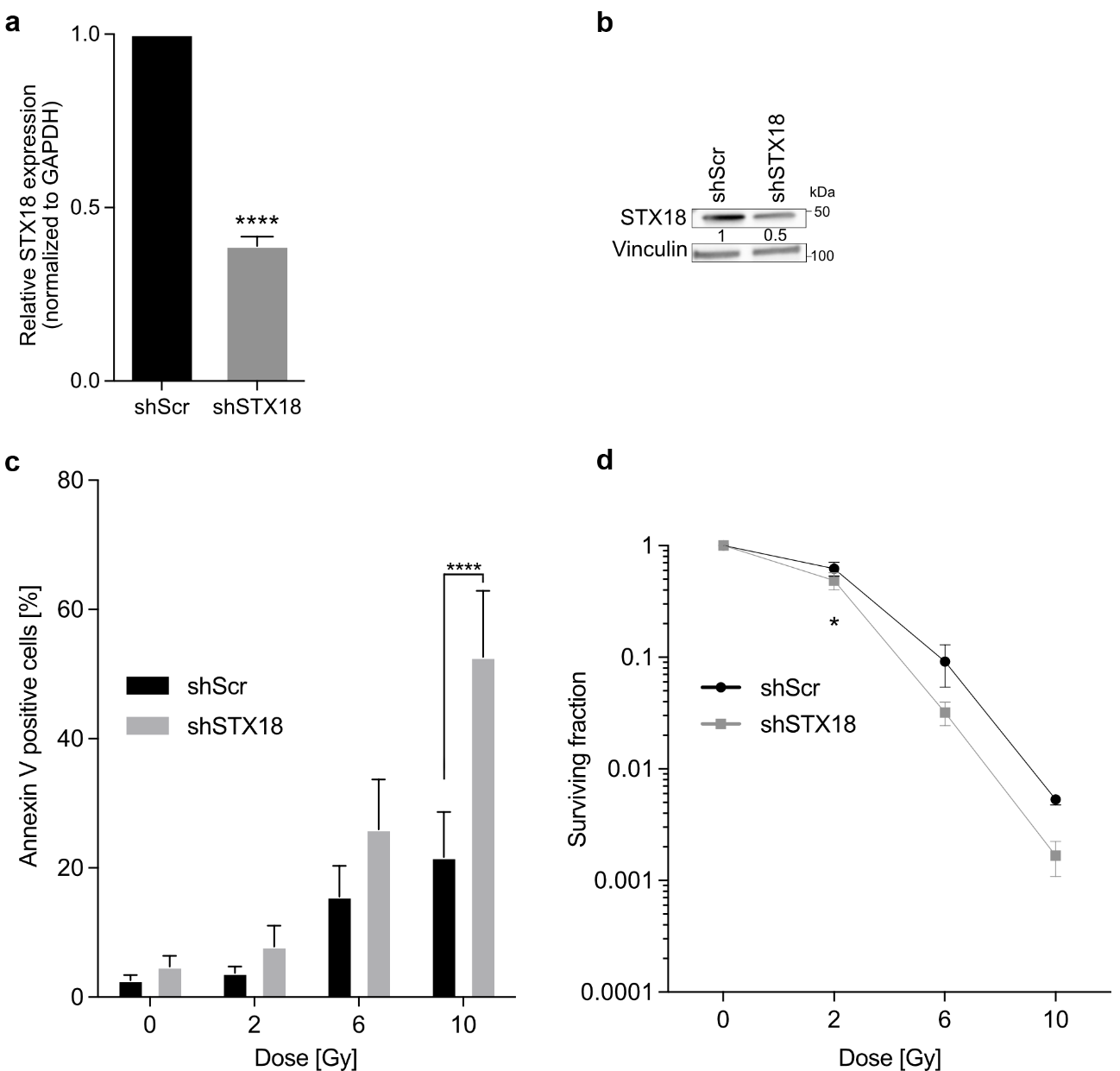


**Supplementary figure 3. A second STX18 shRNA confirms the radiosensitivity of A549-STX18 knockdown cells.**

**a.** A549 cells were transduced with a second shRNA targeting STX18 and its expression was quantified by RT-qPCR. **b.** Immunoblotting analysis of STX18 expression. Vinculin was used as control. After normalization to the loading control, the sample was compared to the shScr sample (set to 1). **c.** Detection by flow cytometry of Annexin V positive cells following irradiation. Cells were incubated for 72 h after irradiation and stained with Annexin V. **d.** Colony formation ability was assessed after irradiation. Cells were irradiated and colonies were counted after 10 days. n=3 for all experiments.


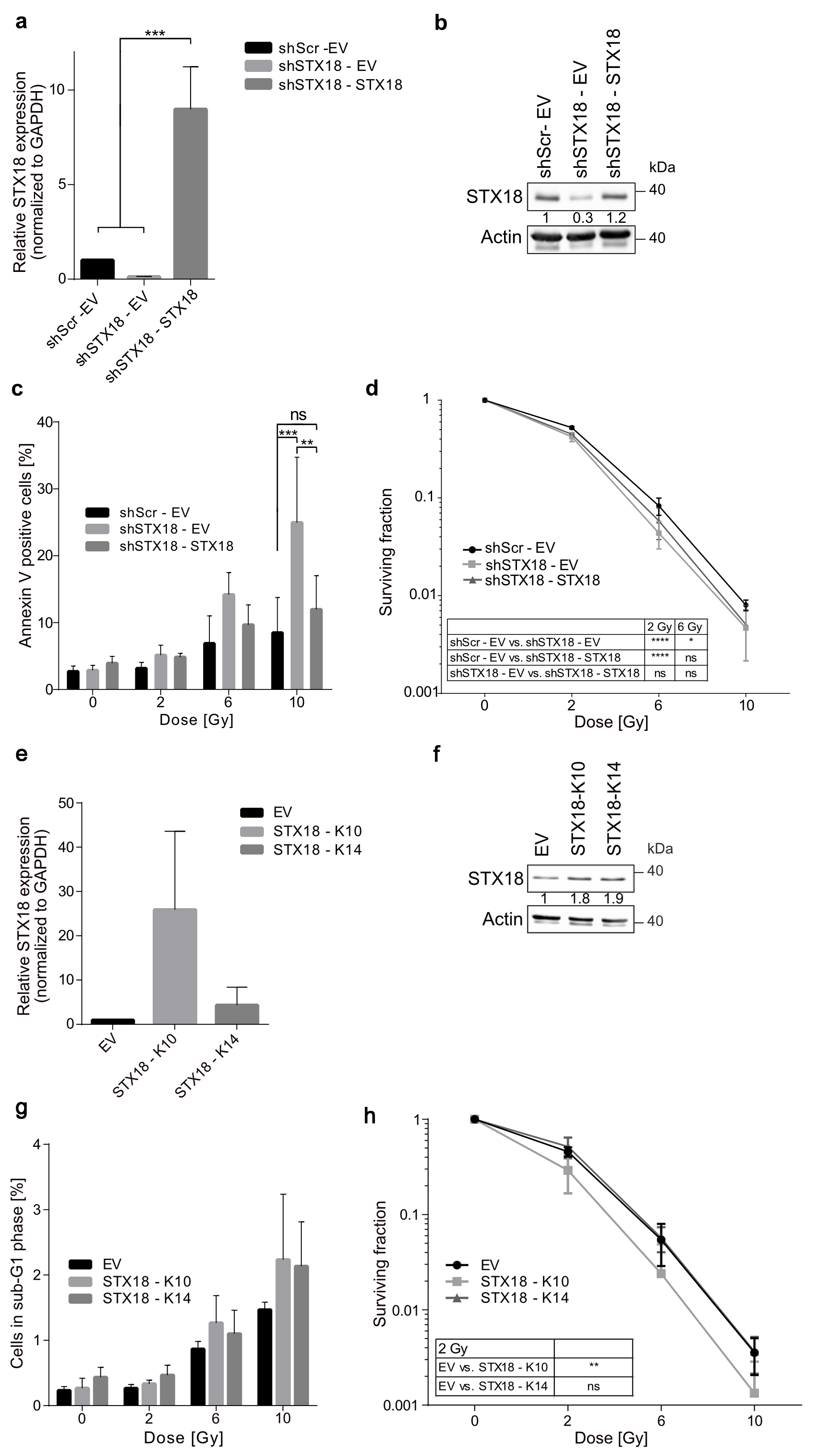


**Supplementary figure 4. Reintroduction of STX18 in A549 cells with STX18 knockdown partially restores resistance to irradiation while its overexpression does not affect radioresistance.**

**a-d.** Syntaxin 18 cDNA or an empty vector (EV) was introduced in A549 shScr and shSTX18 K3 cells.

**a.** RT-qPCR analysis of STX18 mRNA expression. n=3. **b.** Immunoblotting analysis of STX18 expression. Actin was used as control. After normalization to the loading control, the samples were compared to the shScr-EV sample (set to 1). n=3. **c.** Detection by flow cytometry of Annexin V positive cells following irradiation. Cells were incubated for 72 h after irradiation and stained with Annexin V. n=3. **d.** Colony formation ability was assessed after irradiation. Cells were irradiated, and colonies were counted after 10 days. n=3.

**e-h.** A549 parental cells were transduced with a vector encoding the STX18 cDNA or empty vector (EV), and single-cell clones were isolated.

**e.** RT-qPCR analysis of STX18 mRNA expression. n=4. **f.** Immunoblotting analysis of STX18 expression. Actin was used as control. After normalization to the loading control, the samples were compared to the EV sample (set to 1). n=3. **g.** Quantification of sub-G1 fraction by flow cytometry after irradiation. Cells were irradiated and cell cycle distribution was quantified by PI staining after 72 h. n=3. **h.** Colony formation ability was assessed after irradiation. Cells were irradiated and colonies were counted after 10 days. n=3.

**
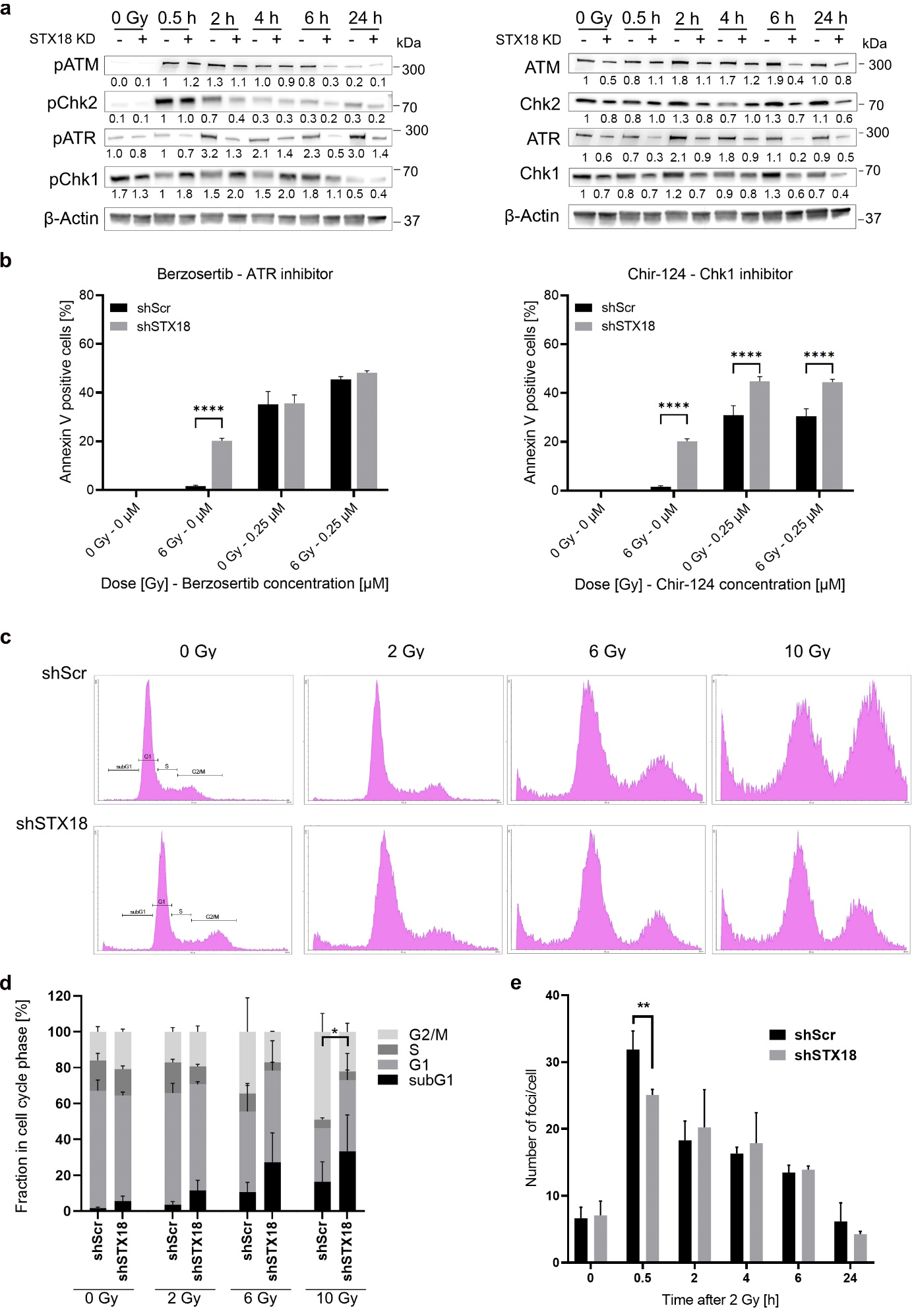
**

**Supplementary figure 5. STX18 knockdown leads to a defect in G2/M checkpoint and reduced DNA damage recognition in line with reduced ATM/ATR activation in H460 cells.**

**a.** Immunoblotting analysis of proteins involved in cell cycle checkpoints after 10 Gy. Actin was used as control. After normalization to the loading control, the samples were compared to 0.5 h shScr for the phosphorylation samples and to untreated shScr for total proteins. For representation of STX18 knockdown, H460-shSTX18 K9 cells were used. **b.** Detection by flow cytometry of Annexin V positive cells following irradiation with 6 Gy and/or pre-treatment with 0.25 µM berzosertib or Chir-124. Cells were incubated for 72 h then stained with Annexin V. **c.** Cell cycle analysis by flow cytometry after irradiation. Cells were irradiated and cell cycle distribution was analyzed by PI staining after 72 h. **d.** Cell cycle analysis 72 h after irradiation. Cells were irradiated and cell cycle distribution was quantified by PI staining. For analysis, the percentage of cells in G2 phase was compared between samples. **e.** Immunofluorescence analysis of γH2A.X foci after irradiation with 2 Gy in H460. The number of foci per cell is represented.


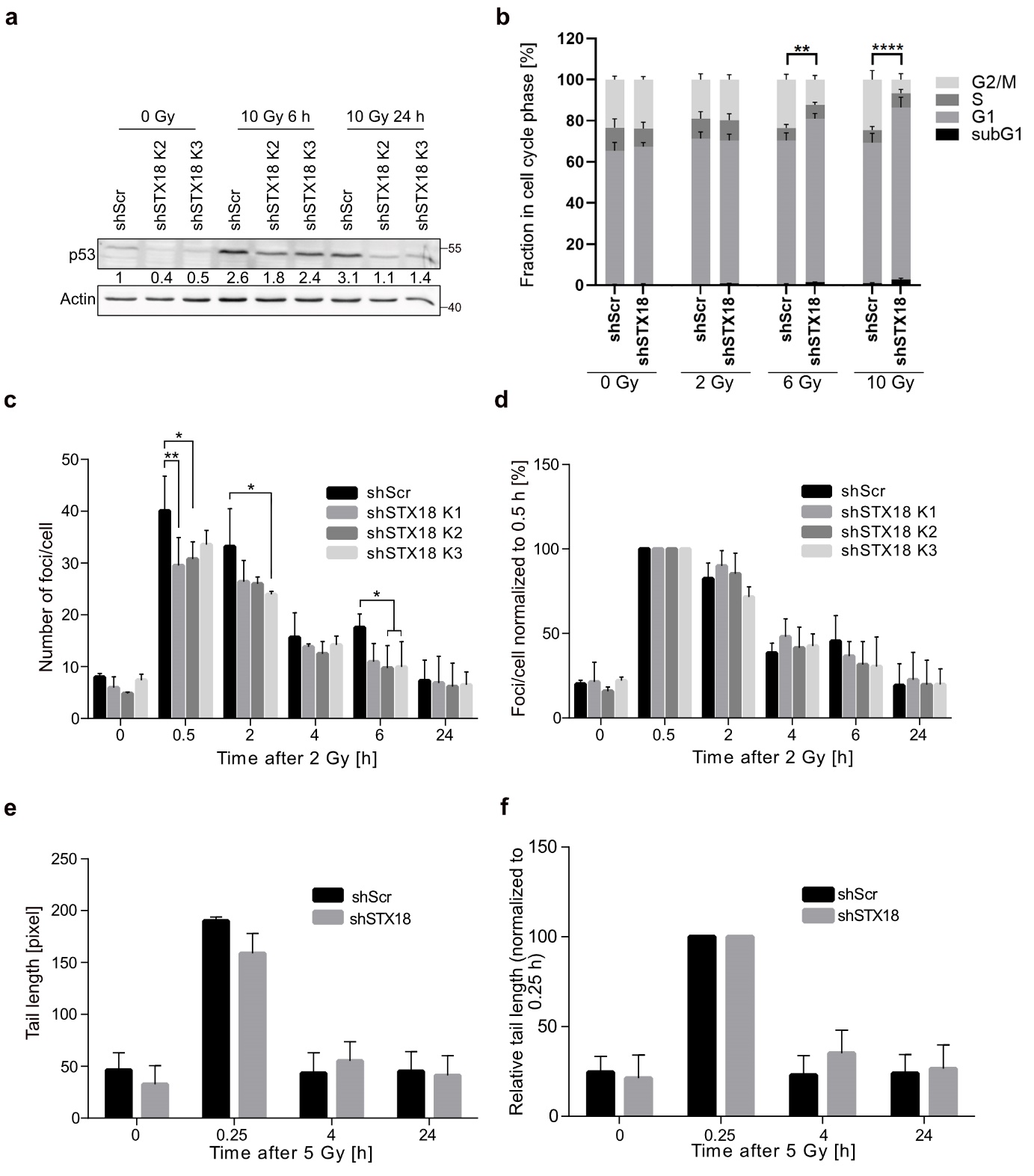


**Supplementary figure 6. Effect of STX18 knockdown on DNA repair efficiency of A549 cells after irradiation.**

**a.** Immunoblotting analysis of p53 after 10 Gy. Actin was used as control. After normalization to the loading control, the samples were compared to the shScr unirradiated sample (set to 1). **b.** Cell cycle analysis by flow cytometry 72 h after irradiation. Cells were irradiated and cell cycle distribution was quantified by PI staining. For analysis, the percentage of cells in G2 phase was compared between samples. For representation of STX18 knockdown, A549-shSTX18 K3 cells were used. **c.** Immunofluorescence analysis of γH2A.X foci after irradiation with 2 Gy in A549. The number of foci per cell is represented. **d.** Immunofluorescence analysis of γH2A.X foci after irradiation with 2 Gy in A549. The number of foci per cell was normalized to the number of foci counted in the 0.5 h sample for each cell line. **e.** Alkaline comet assay after irradiation with 5 Gy. The tail length was quantified by ImageJ. n=3. For representation of STX18 knockdown, A549-shSTX18 K3 cells were used. **f.** Alkaline comet assay after irradiation with 5 Gy. The tail length of each cell was normalized to the tail length measured for the 0.5 h sample for each cell line. n=3 for all experiments.
