# Supplementary material for: Syntaxin 18 regulates the DNA damage response and epithelial-to-mesenchymal transition to promote radiation resistance of lung cancer
Source: Cell Death Dis. 2022 Jun 6;13(6):529. doi: 10.1038/s41419-022-04978-4 (PMC9170725; doi:10.1038/s41419-022-04978-4)
Supplement: Supplementary file 2 — Supplementary Table 1 [file 41419_2022_4978_MOESM2_ESM.pdf]

Gene symbol	Gene name
LPHN1	Adhesion G Protein-Coupled Receptor L1
PLA1A	Phospholipase A1 Member A
RPS6	Ribosomal Protein S6
KIF11	Kinesin Family Member 11
BUB1B	BUB1 Mitotic Checkpoint Serine/Threonine Kinase B
HEXB	Hexosaminidase Subunit Beta
POLR2F	RNA Polymerase II, I And III Subunit F
RPL4	Ribosomal Protein L4
B3GAT3	Beta-1,3-Glucuronyltransferase 3
XCL1	X-C Motif Chemokine Ligand 1
FOXC2	Forkhead Box C2
DUOX1	Dual Oxidase 1
RPS13	Ribosomal Protein S13
STX16	Syntaxin 16
PSMA5	Proteasome 20S Subunit Alpha 5
GPR39	G Protein-Coupled Receptor 39
B3GAT3	Beta-1,3-Glucuronyltransferase 3
CXCL13	C-X-C Motif Chemokine Ligand 13
WIPI1	WD Repeat Domain, Phosphoinositide Interacting 1
PSMA1	Proteasome 20S Subunit Alpha 1
HCLS1	Hematopoietic Cell-Specific Lyn Substrate 1
PRMT5	Protein Arginine Methyltransferase 5
LOC391656	
SMARCC1	SWI/SNF Related, Matrix Associated, Actin Dependent Regulator Of Chromatin Subfamily C Member 1
DDX18	DEAD-Box Helicase 18
DDX47	DEAD-Box Helicase 47
MTHFD1L	Methylenetetrahydrofolate Dehydrogenase (NADP+ Dependent) 1 Like
LOC400652	
CES1	Carboxylesterase 1
WNT5B	Wnt Family Member 5B
THBS2	Thrombospondin 2
LOC391656	
PSMB4	Proteasome 20S Subunit Beta 4
PSMA1	Proteasome 20S Subunit Alpha 1
METTL2B	Methyltransferase 2B, Methylcytidine
EN1	Engrailed Homeobox 1
LOC285053	
NRG3	Neuregulin 3
PSMD13	Proteasome 26S Subunit, Non-ATPase 13
ABCA1	ATP Binding Cassette Subfamily A Member 1
ITSN1	Intersectin 1
CASP7	Caspase 7
RPA1	Replication Protein A1
GRB2	Growth Factor Receptor Bound Protein 2
GUCA1A	Guanylate Cyclase Activator 1A
ARPC2	Actin Related Protein 2/3 Complex Subunit 2
G0S2	G0/G1 Switch 2
RPS13	Ribosomal Protein S13
PSMB6	Proteasome 20S Subunit Beta 6
RUVBL1	RuvB Like AAA ATPase 1
INPP5D	Inositol Polyphosphate-5-Phosphatase D
SREBF2	Sterol Regulatory Element Binding Transcription Factor 2
FABP1	Fatty Acid Binding Protein 1
IRF2	Interferon Regulatory Factor 2
PLA2G4B	Phospholipase A2 Group IVB
STX18	Syntaxin 18
GSK3A	Glycogen Synthase Kinase 3 Alpha
NT5C2	5'-Nucleotidase, Cytosolic II
PCYT1B	Phosphate Cytidylyltransferase 1B, Choline
SH2B2	SH2B Adaptor Protein 2
FZD5	Frizzled 5
