# Supplementary material for: Syntaxin 18 regulates the DNA damage response and epithelial-to-mesenchymal transition to promote radiation resistance of lung cancer
Source: Cell Death Dis. 2022 Jun 6;13(6):529. doi: 10.1038/s41419-022-04978-4 (PMC9170725; doi:10.1038/s41419-022-04978-4)
Supplement: Supplementary file 3 — Supplementary Table 2 [file 41419_2022_4978_MOESM3_ESM.docx]

**Supplementary table 2. List of shRNA used for RNA interference**. All shRNA were purchased from Sigma-Aldrich.

| **Gene target** | **Name** | **Sequence (5’-3’)** |
| --- | --- | --- |
| Scrambled control | shScr | CCGGCAACAAGATGAAGAGCACCAACTCGAGTTGGTGCTCTTCATCTTGTTGTTTTT |
| *STX18* | shSTX18 | CCTAATTGTCTCAGGGTTCAA |
| *STX18* | shSTX18 (2^nd^) | GACATAAGAGAGGCCATTAAA |
| *FZD5* | shFZD5 | GCGTTTCTTACTGCCTTCTTT |
