# Supplementary material for: Syntaxin 18 regulates the DNA damage response and epithelial-to-mesenchymal transition to promote radiation resistance of lung cancer
Source: Cell Death Dis. 2022 Jun 6;13(6):529. doi: 10.1038/s41419-022-04978-4 (PMC9170725; doi:10.1038/s41419-022-04978-4)
Supplement: Supplementary file 4 — Supplementary Table 3 [file 41419_2022_4978_MOESM4_ESM.docx]

**Supplementary table 3. List of human primers for RT-qPCR**. List of primers and their sequences.

| **Name** | **Sequence (3‘-5‘)** | **Source** |
| --- | --- | --- |
| *GAPDH* | For ATTGCCCTCAACGACCACT | Custom DNA oligos - IDT |
|  | Rev TCTTCCTCTTGTGCTCTTGCT |  |
| *MMP9* | For GCACGACGTCTTCCAGTACC | Safranek et al. 2009 (1) |
|  | Rev CAGGATGTCATAGGTCACGTA GC |  |
| *STX18* | For TTGAAGGGAAAGGAGTTCAG | Custom DNA oligos - IDT |
|  | Rev CTGCATTCAGTCTGTCTTGC |  |

**Supplementary references**

1. Safranek J, Pesta M, Holubec L, Kulda V, Dreslerova J, Vrzalova J, et al. Expression of MMP-7, MMP-9, TIMP-1 and TIMP-2 mRNA in lung tissue of patients with non-small cell lung cancer (NSCLC) and benign pulmonary disease. Anticancer Res. 2009;29(7):2513-7.
