# Supplementary material for: Syntaxin 18 regulates the DNA damage response and epithelial-to-mesenchymal transition to promote radiation resistance of lung cancer
Source: Cell Death Dis. 2022 Jun 6;13(6):529. doi: 10.1038/s41419-022-04978-4 (PMC9170725; doi:10.1038/s41419-022-04978-4)
Supplement: Supplementary file 5 — Supplementary Table 4 [file 41419_2022_4978_MOESM5_ESM.pdf]

Figure 2a A549
STX18

Sample	Mean	SD	Significance	
shScr	1,00			
shSTX18 K1	0,72	0,17	ns	vs shScr
shSTX18 K2	0,53	0,31	*	vs shScr
shSTX18 K3	0,24	0,16	**	vs shScr

Figure 2a H460
STX18

Sample	Mean	SD	Significance	
shScr	1.0			
shSTX18 K1	0.76	0,05	*	vs shScr
shSTX18 K2	0,61	0,12	**	vs shScr
shSTX18 K3	0,90	0,10	ns	vs shScr

Figure 4a
ATM

	shScr		shSTX18			
Sample	Mean	SD	Mean	SD	Significance	
0	0,65	0,21	0,45	0,22	ns	vs shScr 0
0,5	1,00		0,72	0,15	no test	vs shScr 0.5
2	1,56	0,53	1,02	0,54	ns	vs shScr 2
4	1,31	0,39	0,72	0,33	ns	vs shScr 4
6	1,48	0,60	0,45	0,29	**	vs shScr 6
24	0,88	0,48	0,37	0,02	ns	vs shScr 24

pATM

	shScr		shSTX18			
Sample	Mean	SD	Mean	SD	Significance	
0	0,01	0,01	0,01	0,01	ns	vs shScr 0
0,5	1,00		0,48	0,07	no test	vs shScr 0.5
2	1,25	0,16	0,53	0,09	****	vs shScr 2
4	1,24	0,42	0,39	0,04	****	vs shScr 4
6	0,74	0,09	0,22	0,06	***	vs shScr 6
24	0,30	0,11	0,12	0,07	ns	vs shScr 24

Chk2

	shScr		shSTX18			
Sample	Mean	SD	Mean	SD	Significance	
0	1,41	0,87	1,73	0,58	ns	vs shScr 0
0,5	1,00		1,41	0,38	no test	vs shScr 0.5
2	1,60	1,05	1,54	0,65	ns	vs shScr 2
4	1,41	0,73	1,98	0,98	ns	vs shScr 4
6	2,84	2,43	1,68	0,65	ns	vs shScr 6
24	1,29	0,20	1,00	0,36	ns	vs shScr 24

pChk2

	shScr		shSTX18			
Sample	Mean	SD	Mean	SD	Significance	
0	0,07	0,03	0,11	0,08	ns	vs shScr 0
0,5	1,00		0,78	0,05	no test	vs shScr 0.5
2	1,76	1,14	1,79	0,82	ns	vs shScr 2
4	1,16	0,24	1,69	0,36	ns	vs shScr 4
6	1,01	0,44	1,49	0,62	ns	vs shScr 6
24	0,67	0,45	0,56	0,35	ns	vs shScr 24

ATR

	shScr		shSTX18			
Sample	Mean	SD	Mean	SD	Significance	
0	0,74	0,11	0,81	0,06	ns	vs shScr 0
0,5	1,00		0,97	0,51	no test	vs shScr 0.5
2	1,30	0,26	1,11	0,63	ns	vs shScr 2
4	1,67	0,77	0,96	0,36	*	vs shScr 4
6	1,49	0,51	0,77	0,20	*	vs shScr 6
24	0,96	0,32	0,49	0,30	ns	vs shScr 24

pATR

	shScr		shSTX18			
Sample	Mean	SD	Mean	SD	Significance	
0	0,25	0,20	0,17	0,14	ns	vs shScr 0
0,5	1,00		0,55	0,27	no test	vs shScr 0.5
2	3,51	0,85	1,63	0,53	ns	vs shScr 2
4	5,00	2,07	2,57	1,67	ns	vs shScr 4
6	6,30	2,90	2,17	1,12	**	vs shScr 6
24	3,39	2,16	2,19	2,94	ns	vs shScr 24

Chk1

	shScr		shSTX18			
Sample	Mean	SD	Mean	SD	Significance	
0	1,07	0,38	0,75	0,18	ns	vs shScr 0
0,5	1,00		0,72	0,20	no test	vs shScr 0.5
2	1,06	0,01	0,73	0,22	ns	vs shScr 2
4	1,28	0,38	0,72	0,34	**	vs shScr 4
6	1,05	0,21	0,61	0,10	*	vs shScr 6
24	0,56	0,07	0,43	0,21	ns	vs shScr 24

pChk1

	shScr		shSTX18			
Sample	Mean	SD	Mean	SD	Significance	
0	0,20	0,23	0,15	0,18	ns	vs shScr 0
0,5	1,00		0,76	0,35	no test	vs shScr 0.5
2	0,88	0,24	0,40	0,30	ns	vs shScr 2
4	0,73	0,39	0,42	0,37	ns	vs shScr 4
6	0,68	0,40	0,44	0,36	ns	vs shScr 6
24	0,36	0,30	0,40	0,47	ns	vs shScr 24

Figure 5a
E-cadherin

Sample	Mean	SD	Significance	
shScr	1,00			
shSTX18 K1	3,10	0,84	**	vs shScr
shSTX18 K2	3,10	0,60	**	vs shScr
shSTX18 K3	3,50	0,36	**	vs shScr

Vimentin

Sample	Mean	SD	Significance	
shScr	1,00			
shSTX18 K1	0,37	0,29	*	vs shScr

shSTX18 K2	0,36	0,26	*	vs shScr
shSTX18 K3	0,16	0,12	**	vs shScr

Zeb1

Sample	Mean	SD	Significance	
shScr	1,00			
shSTX18 K1	0,51	0,20	ns	vs shScr
shSTX18 K2	0,49	0,33	ns	vs shScr
shSTX18 K3	0,79	0,63	ns	vs shScr

ZO1

Sample	Mean	SD	Significance	
shScr	1,00			
shSTX18 K1	0,82	0,19	ns	vs shScr
shSTX18 K2	1,00	0,22	ns	vs shScr
shSTX18 K3	1,20	0,77	ns	vs shScr

Suppl. fig. 1a

FZD5

Sample	Mean
shScr	1,00
shFZD5	0,18
shFZD5 K15	0,12
shFZD5 K16	0,15

Suppl. fig. 3b

STX18

Sample	Mean
shScr	1,00
shSTX18	0,48

Suppl. fig. 4b

STX18

Sample	Mean	SD	Significance	
shScr - EV	1,00			
shSTX18 - EV	0,29	0,16	*	vs shScr- EV
shSTX18 - STX18	1,20	0,32	**	vs shSTX18-EV

Suppl. fig. 4f

STX18

Sample	Mean	SD	Significance	
EV	1,00			
STX18-K10	1,80	0,74	ns	vs EV
STX18-K14	1,90	0,83	ns	vs EV

Suppl. fig. 5a

ATM

	shScr		shSTX18			
Sample	Mean	SD	Mean	SD	Significance	
0	1,05	0,20	1,76	1,03	ns	vs shScr 0
0,5	1,00		1,51	0,30	no test	vs shScr 0.5
2	1,43	0,70	1,38	0,22	ns	vs shScr 2
4	1,34	0,64	1,71	0,45	ns	vs shScr 4
6	1,66	0,53	0,89	0,41	ns	vs shScr 6
24	1,31	0,08	1,18	0,68	ns	vs shScr 24

pATM

	shScr		shSTX18			
Sample	Mean	SD	Mean	SD	Significance	
0	0,04	0,02	0,18	0,14	ns	vs shScr 0
0,5	1,00		1,83	0,56	no test	vs shScr 0.5
2	1,17	0,13	1,49	0,39	ns	vs shScr 2
4	0,81	0,12	1,33	0,35	*	vs shScr 4
6	0,74	0,07	0,60	0,31	ns	vs shScr 6
24	0,35	0,14	0,43	0,26	ns	vs shScr 24

Chk2

	shScr		shSTX18			
Sample	Mean	SD	Mean	SD	Significance	
0	0,97	0,28	1,54	0,50	ns	vs shScr 0
0,5	1,00		1,56	0,30	no test	vs shScr 0.5
2	0,98	0,55	1,28	0,20	ns	vs shScr 2
4	0,44	0,36	1,59	0,25	**	vs shScr 4
6	1,28	0,36	1,10	0,20	ns	vs shScr 6
24	0,81	0,57	0,69	0,09	ns	vs shScr 24

pChk2

	shScr		shSTX18			
Sample	Mean	SD	Mean	SD	Significance	
0	0,05	0,03	0,11	0,05	ns	vs shScr 0
0,5	1,00		1,58	0,52	no test	vs shScr 0.5
2	0,53	0,13	0,55	0,23	ns	vs shScr 2
4	0,29	0,07	0,46	0,24	ns	vs shScr 4
6	0,35	0,10	0,24	0,08	ns	vs shScr 6
24	0,37	0,09	0,37	0,17	ns	vs shScr 24

ATR

	shScr		shSTX18			
Sample	Mean	SD	Mean	SD	Significance	
0	1,45	0,20	1,59	0,86	ns	vs shScr 0
0,5	1,00		0,72	0,22	no test	vs shScr 0.5
2	1,86	1,05	1,34	0,49	ns	vs shScr 2
4	1,67	0,77	1,57	0,63	ns	vs shScr 4
6	1,47	0,22	0,58	0,30	*	vs shScr 6
24	1,64	0,51	1,19	0,62	*	vs shScr 24

pATR

	shScr		shSTX18			
Sample	Mean	SD	Mean	SD	Significance	
0	1,39	0,42	1,38	0,54	ns	vs shScr 0
0,5	1,00		0,83	0,15	no test	vs shScr 0.5
2	2,48	0,72	1,95	0,58	ns	vs shScr 2
4	2,36	0,29	3,29	1,83	ns	vs shScr 4
6	2,89	0,55	1,01	0,46	ns	vs shScr 6
24	4,48	1,34	2,65	1,77	ns	vs shScr 24

Chk1

	shScr		shSTX18			
**Sample**	**Mean**	**SD**	**Mean**	**SD**	**Significance**	
0	1,02	0,18	1,04	0,15	ns	vs shScr 0
0,5	1,00		1,00	0,16	no test	vs shScr 0.5
2	1,13	0,27	1,02	0,15	ns	vs shScr 2
4	0,90	0,25	1,18	0,18	ns	vs shScr 4
6	1,15	0,39	0,83	0,13	ns	vs shScr 6
24	0,63	0,17	0,55	0,12	ns	vs shScr 24

pChk1

	shScr		shSTX18			
**Sample**	**Mean**	**SD**	**Mean**	**SD**	**Significance**	
0	2,57	0,84	2,66	1,76	ns	vs shScr 0
0,5	1,00		2,95	1,59	no test	vs shScr 0.5
2	1,33	0,31	3,17	2,04	ns	vs shScr 2
4	1,11	0,40	3,58	2,47	*	vs shScr 4
6	1,61	0,34	2,42	1,55	ns	vs shScr 6
24	0,63	0,21	0,88	0,82	ns	vs shScr 24

Suppl. fig. 6a

p53

	shScr		shSTX18 K2			shSTX18 K3			
**Sample**	**Mean**	**SD**	**Mean**	**SD**	**Significance**	**Mean**	**SD**	**Significance**	
0 Gy	1,00		0,41	0,23	ns	0,46	0,22	ns	vs shScr 0 Gy
10 Gy - 6 h	2,56	1,10	1,76	0,75	ns	2,36	1,39	ns	vs shScr 6 h
10 Gy - 24 h	3,13	1,16	1,12	0,45	*	1,42	0,50	*	vs shScr 24 h

After normalization to the loading control, the samples were compared to 0.5 h shScr for the phosphorylation samples and to untreated shScr for total proteins. Significance was calculated using twoway-ANOVA without correction.
