# Supplementary material for: Syntaxin 18 regulates the DNA damage response and epithelial-to-mesenchymal transition to promote radiation resistance of lung cancer
Source: Cell Death Dis. 2022 Jun 6;13(6):529. doi: 10.1038/s41419-022-04978-4 (PMC9170725; doi:10.1038/s41419-022-04978-4)
Supplement: Supplementary file 6 — Uncropped Western Blots [file 41419_2022_4978_MOESM6_ESM.pdf]

Uncropped western blots

The first blot in the row of each antibody represents the blot pictured in the figures of the manuscript and supplementary. The other two were additionally analyzed using densitometry and used for the calculation of the mean of n = 3.

Figure 2a A549

**STX18**	**Actin/Vinculin**
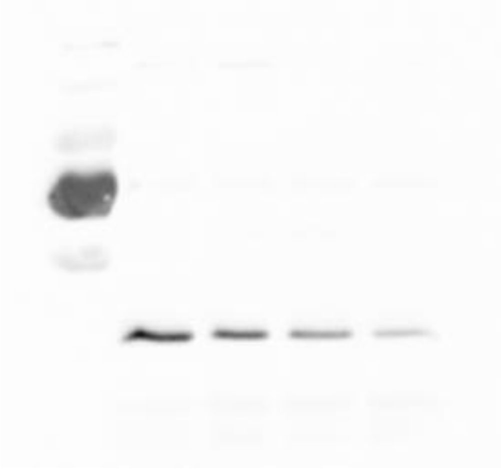	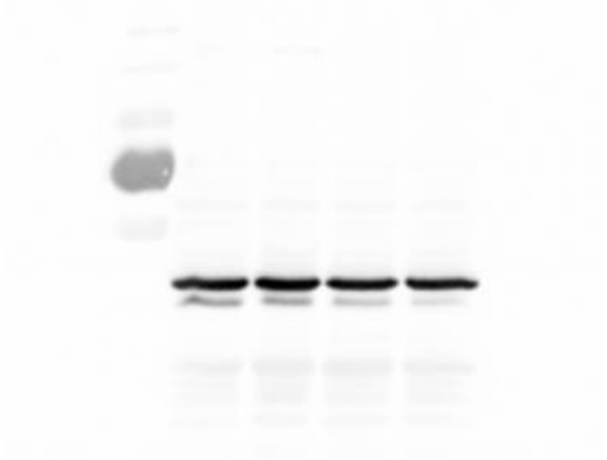
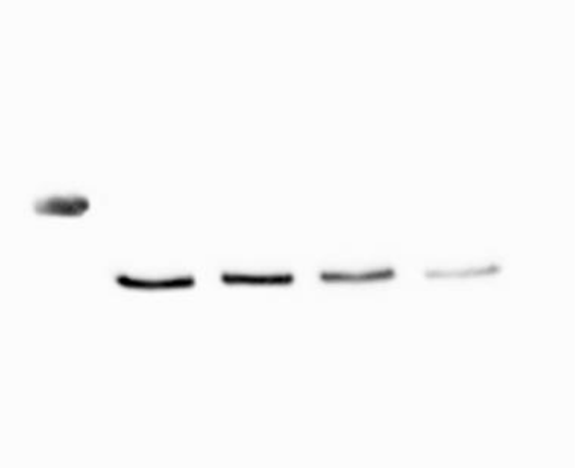	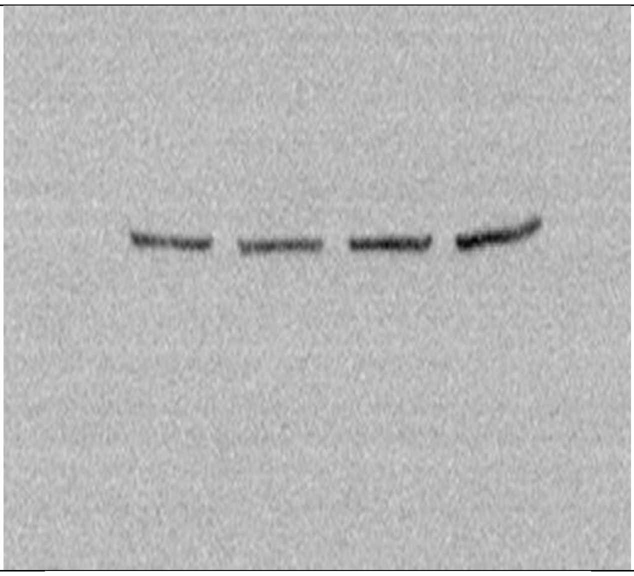
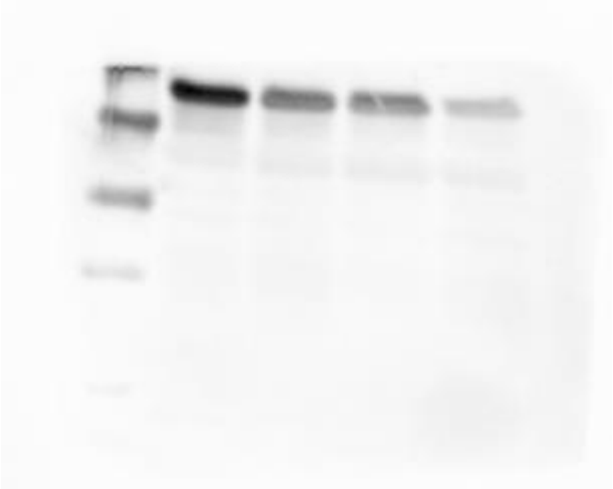	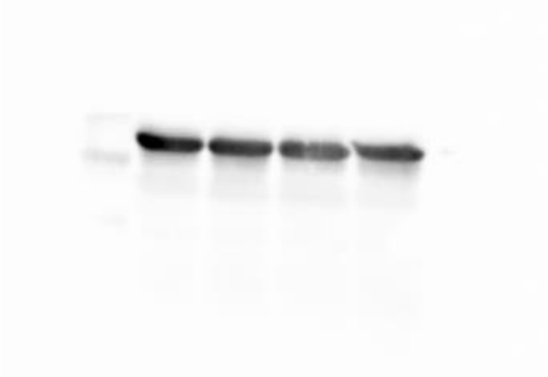

Figure 2a H460

STX18	Actin
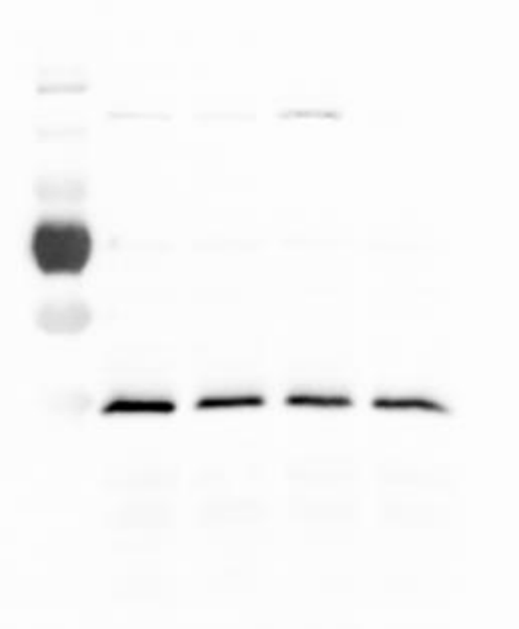	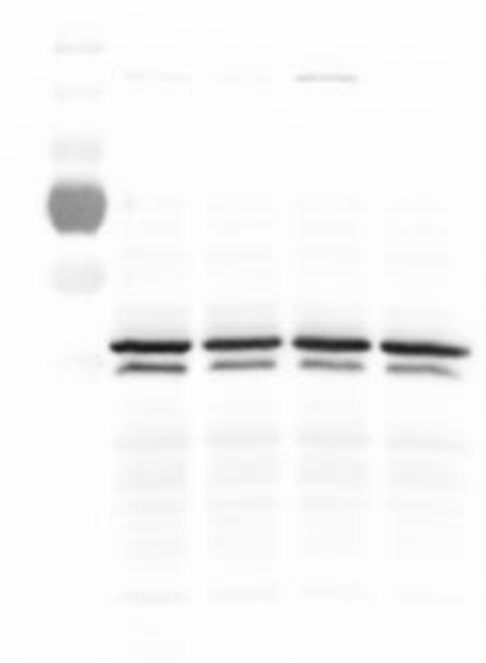
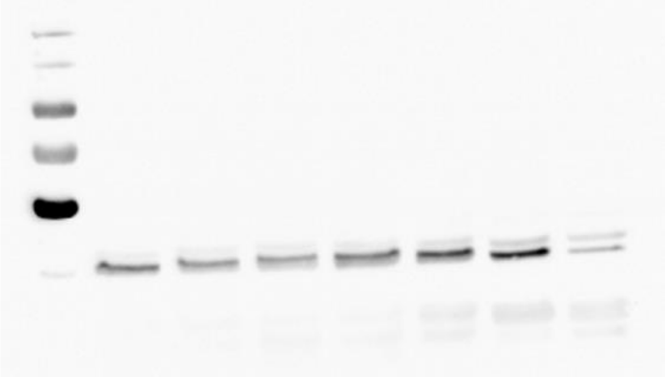	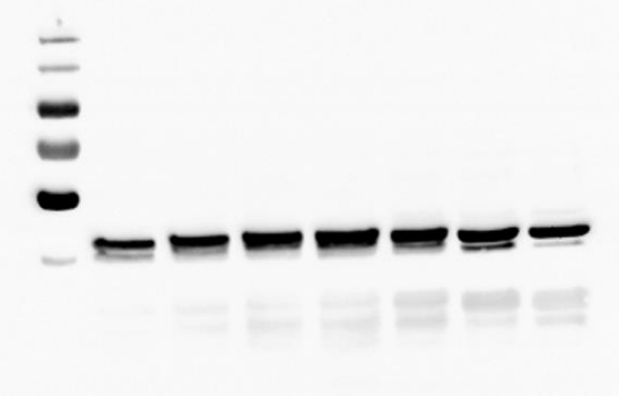
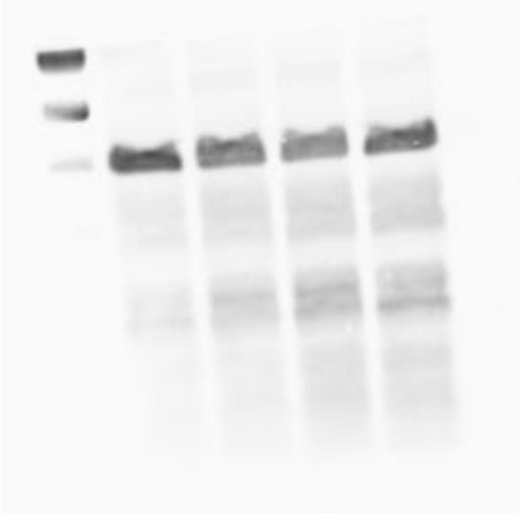	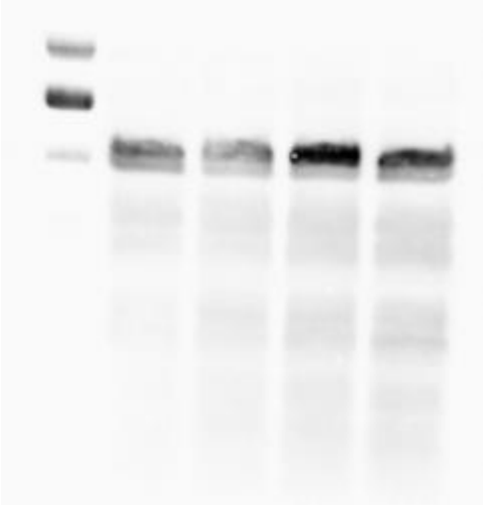

Figure 4a

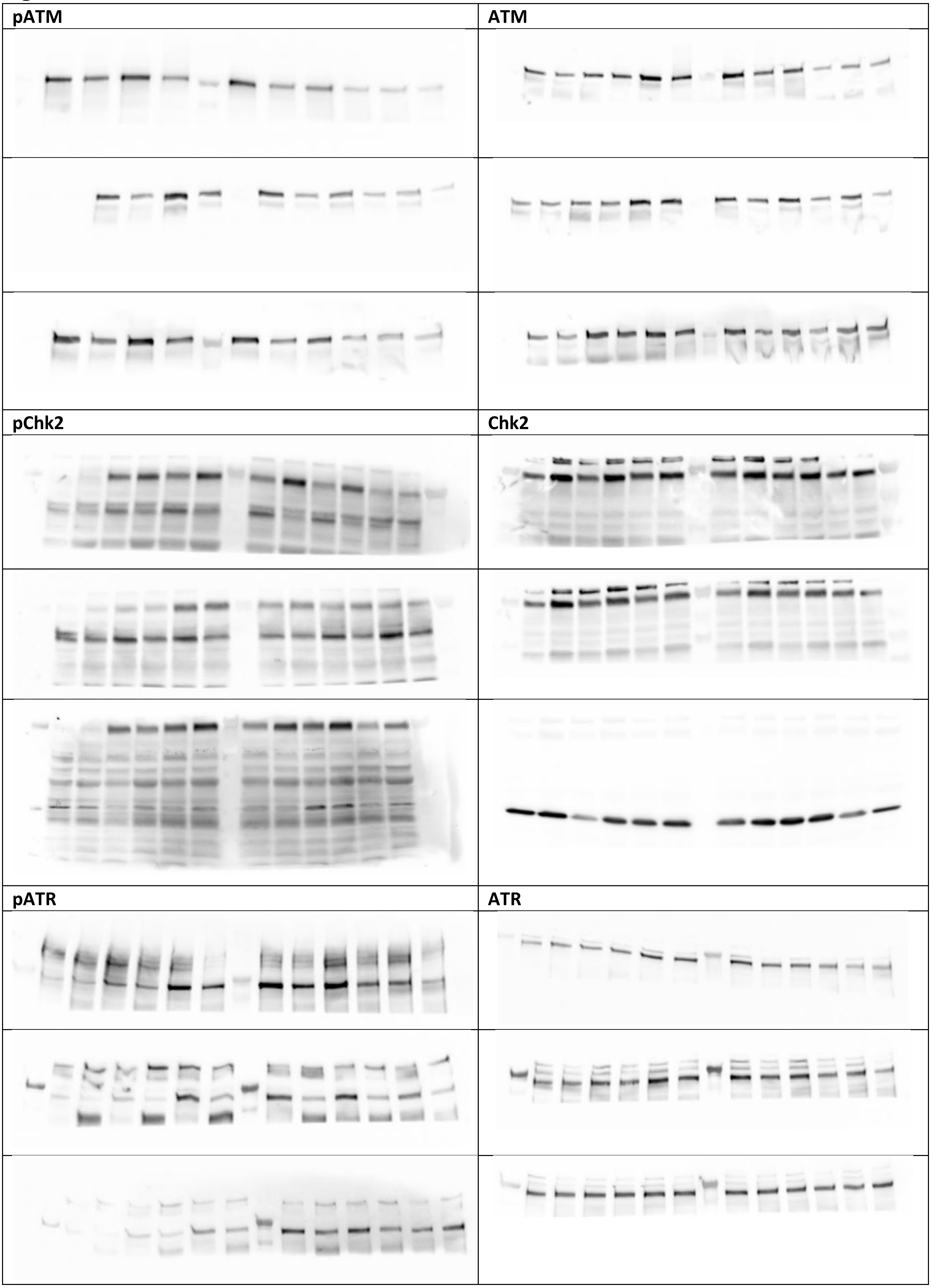

pChk1	Chk1
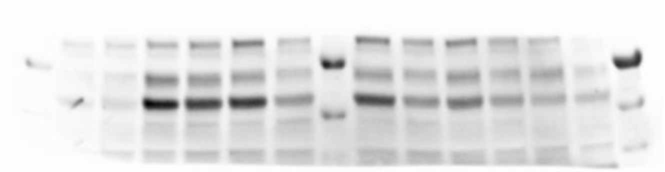	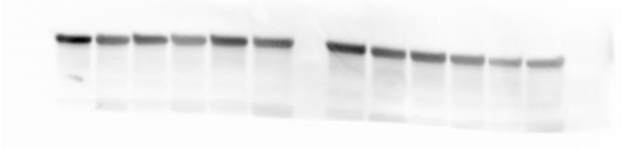
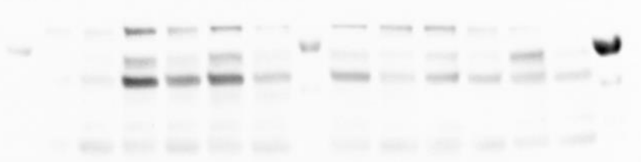	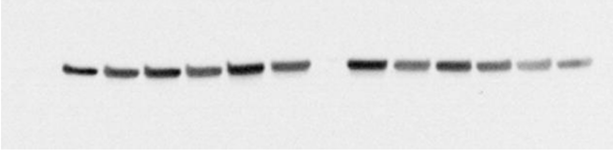
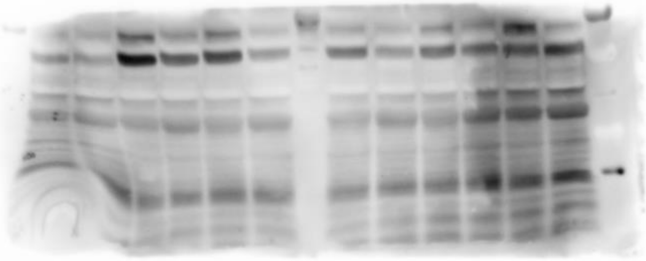	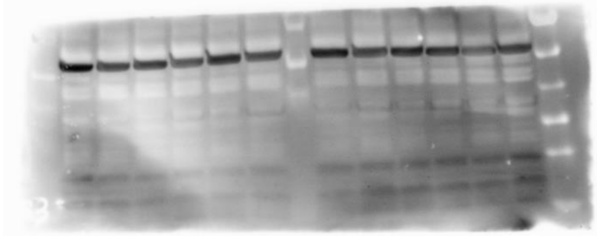
**Vinculin**	**Vinculin**
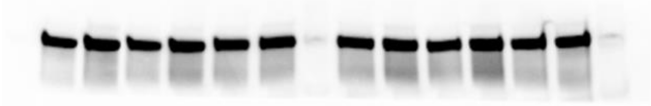	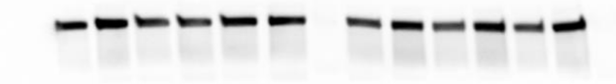
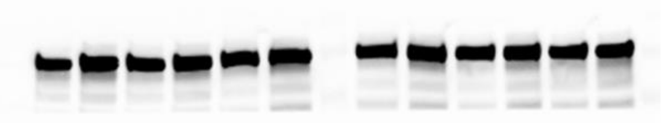	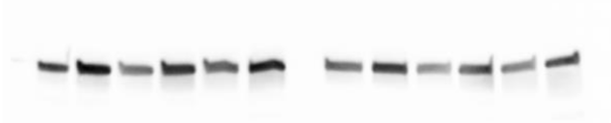
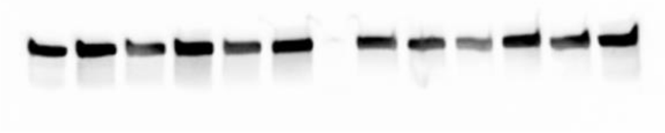	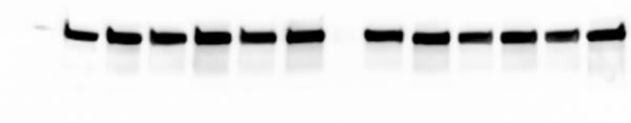

Figure 5a

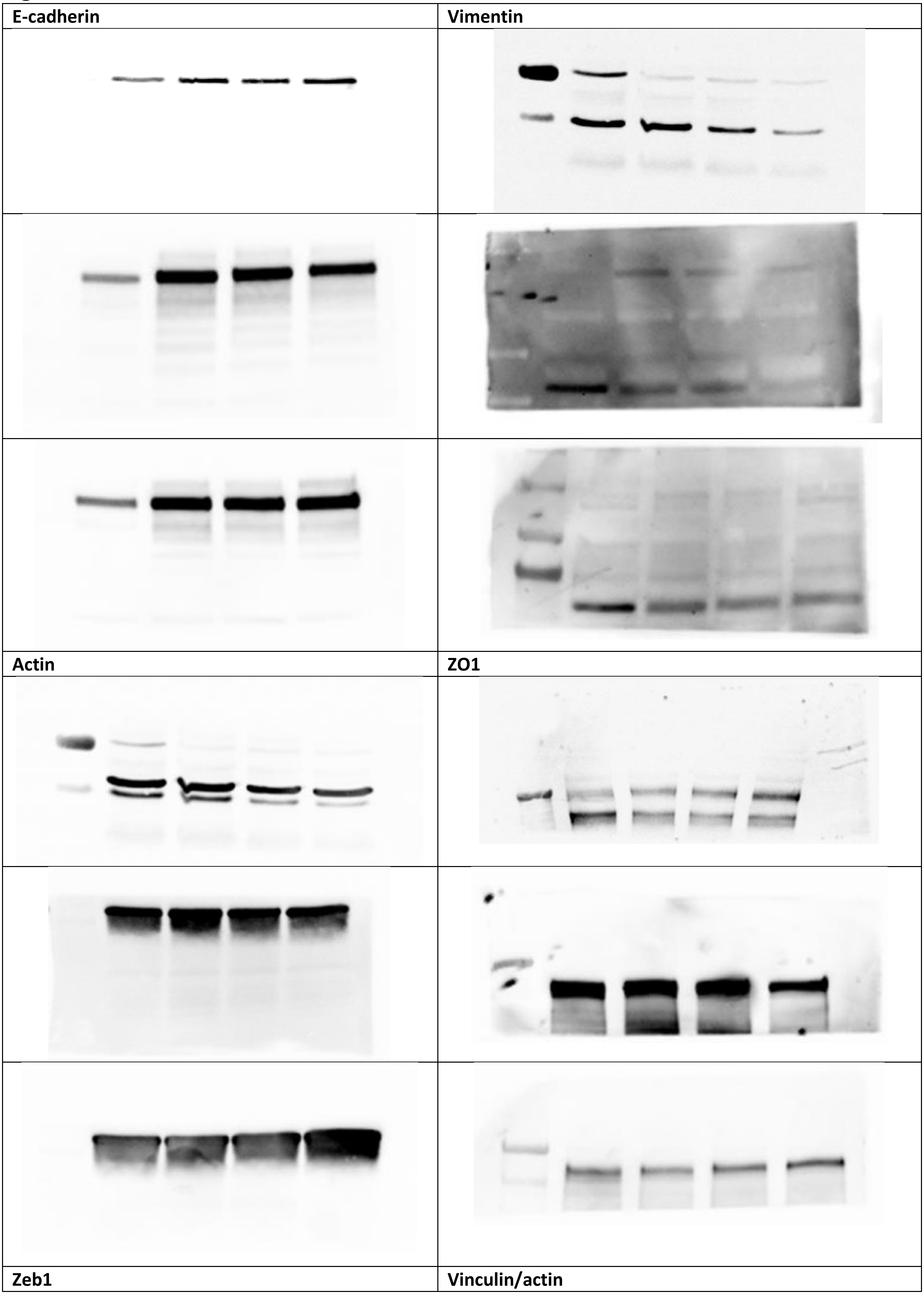

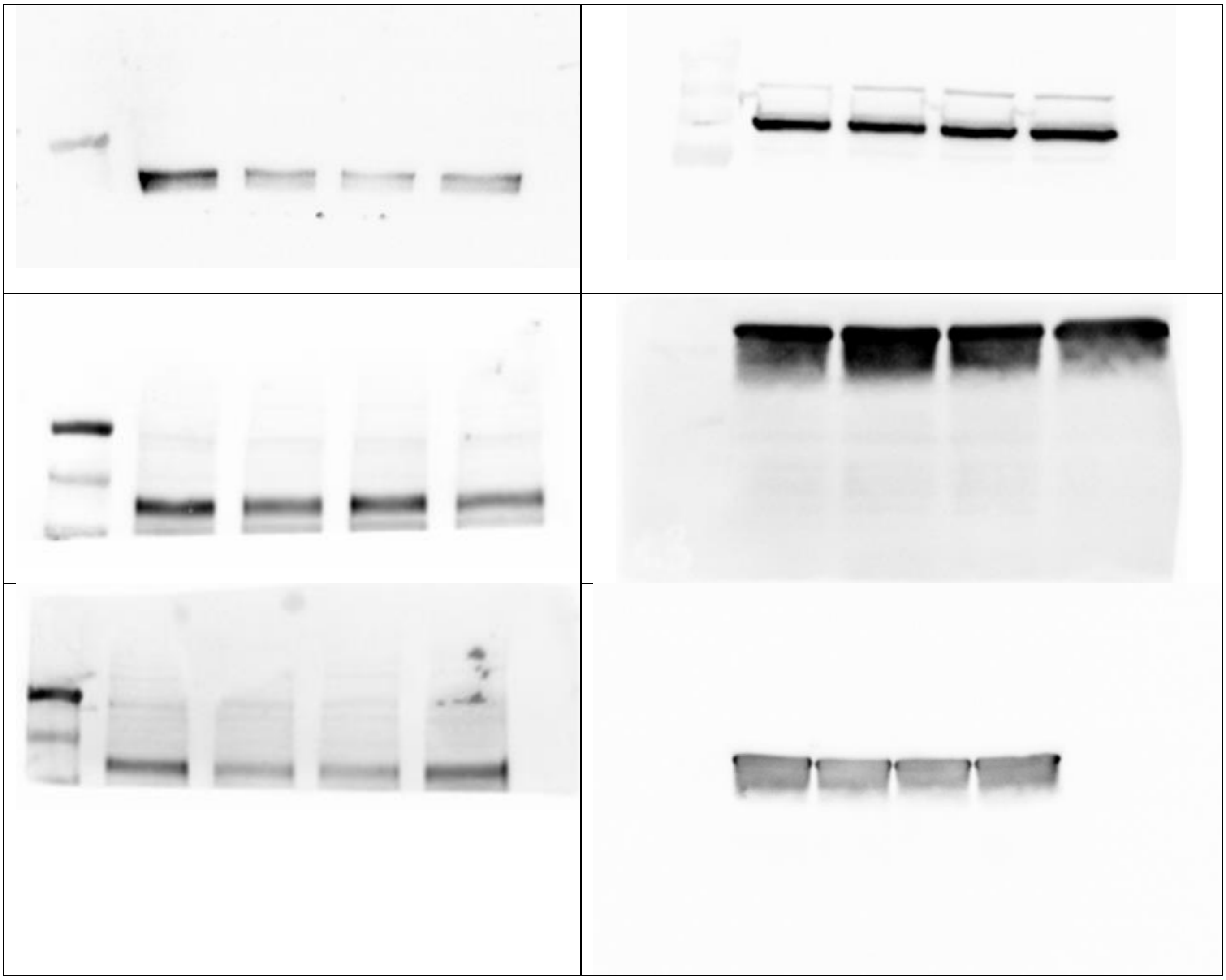

Supplementary figure 1a

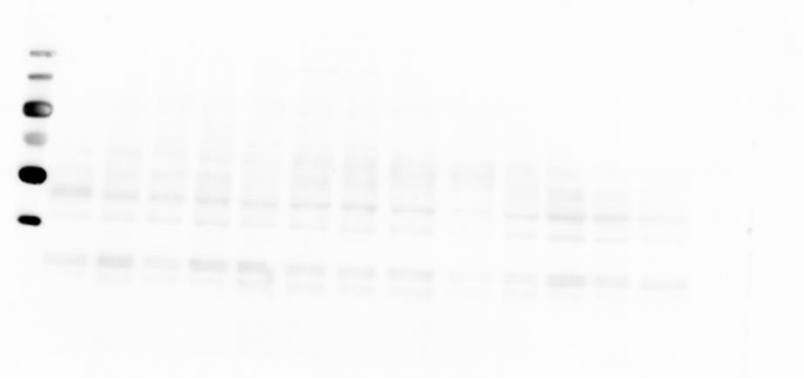

FZD5

Actin

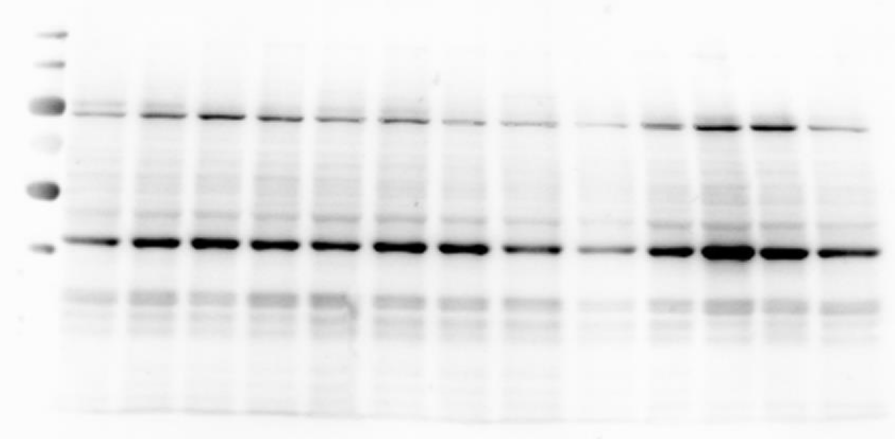

Supplementary figure 3b

STX18	Vinculin
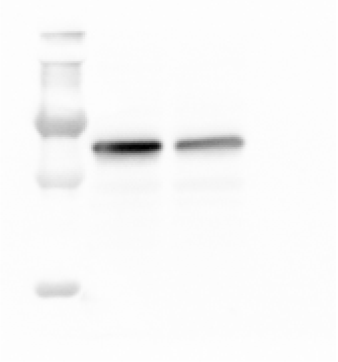	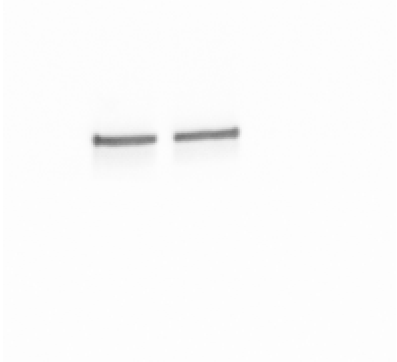
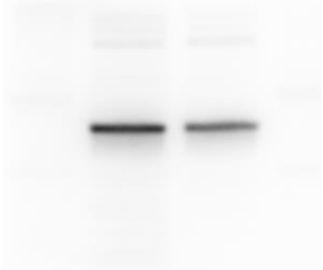	

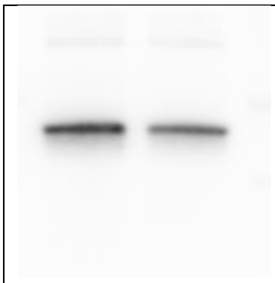	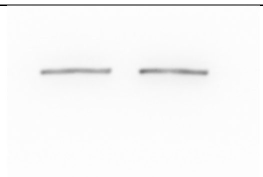

Supplementary figure 4b

STX18	Actin
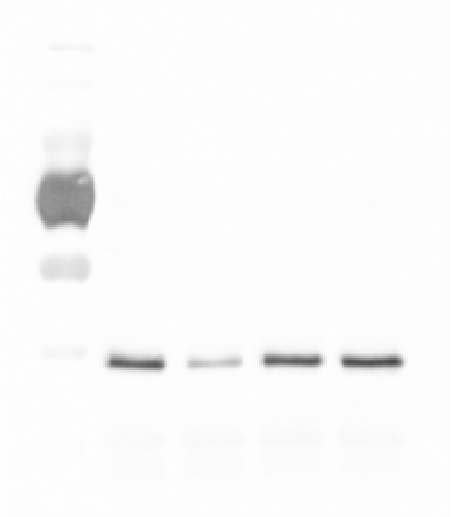	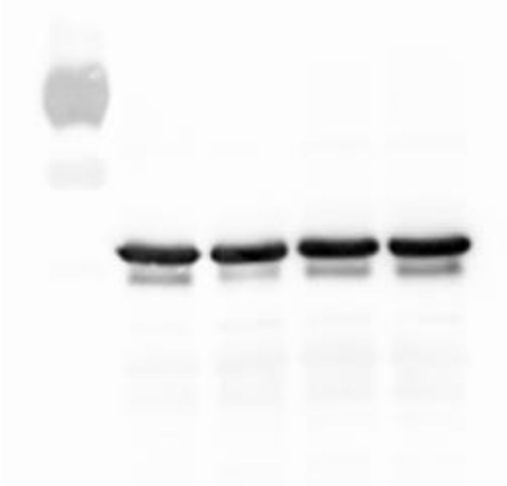
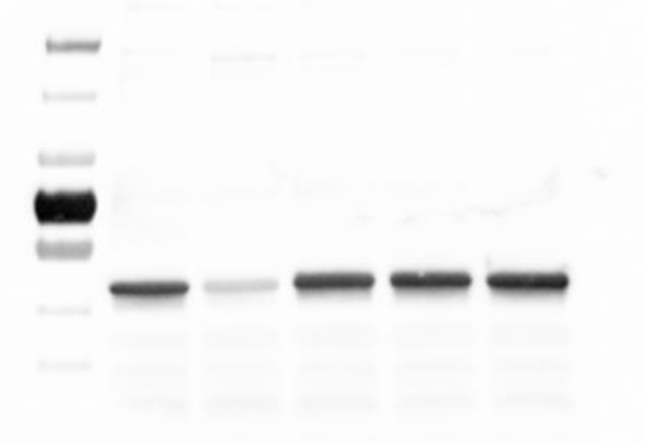	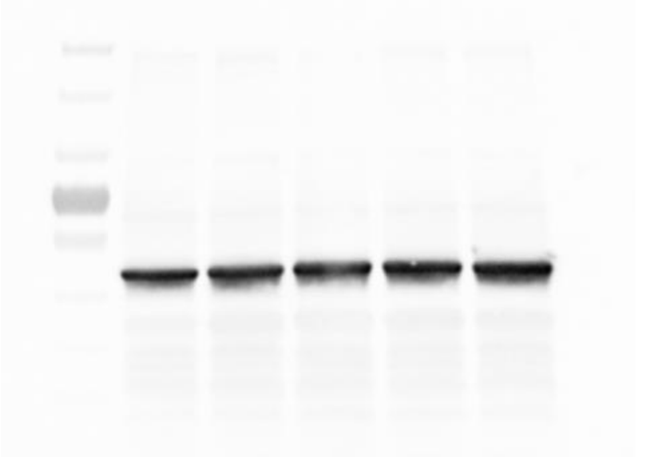

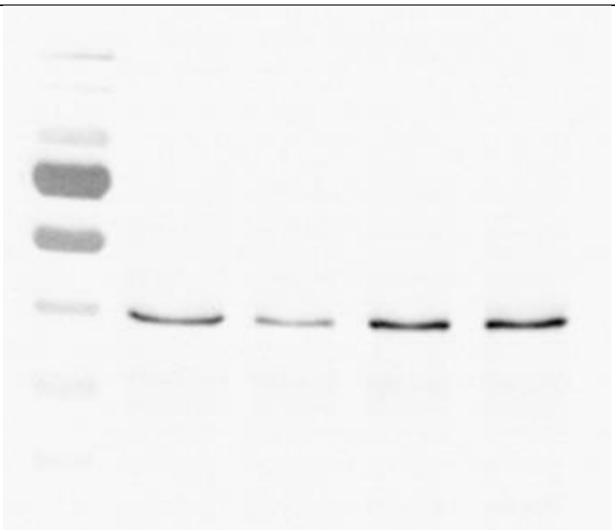	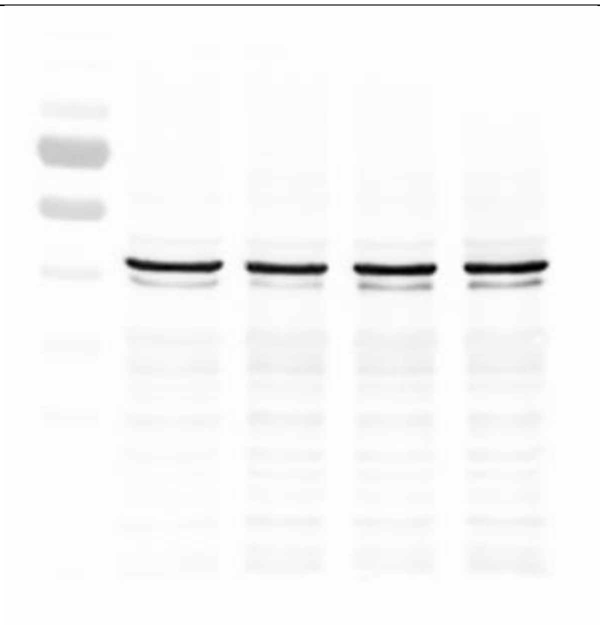

Supplementary figure 4f

STX18	Actin
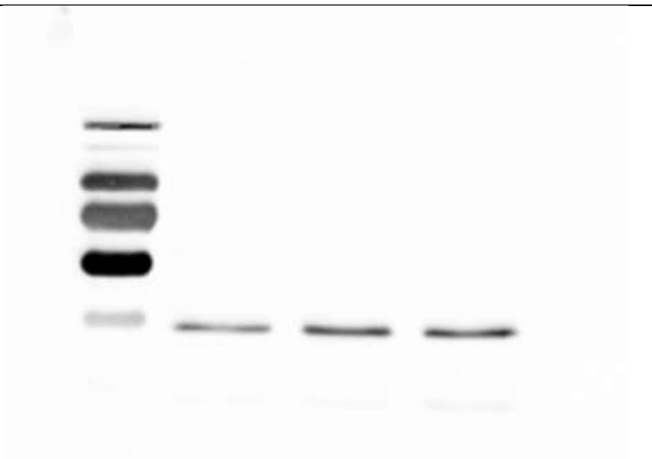	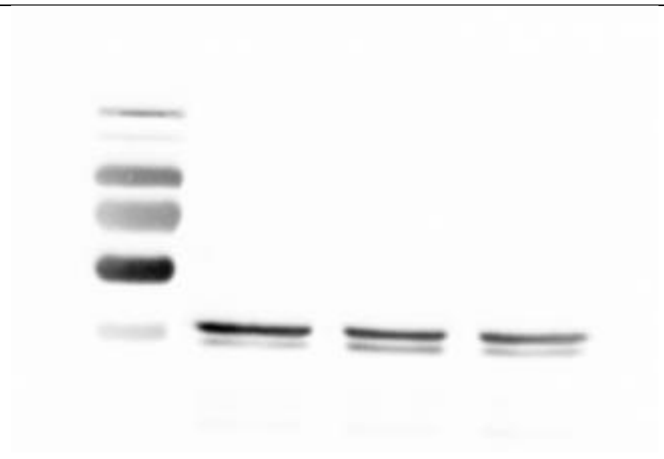
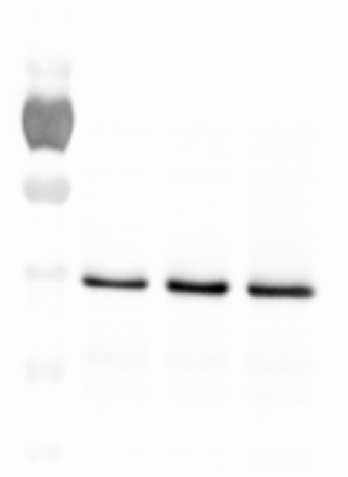	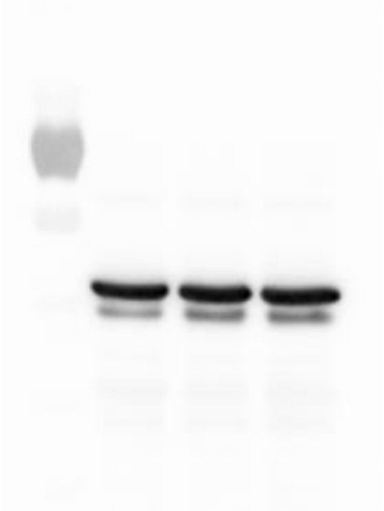

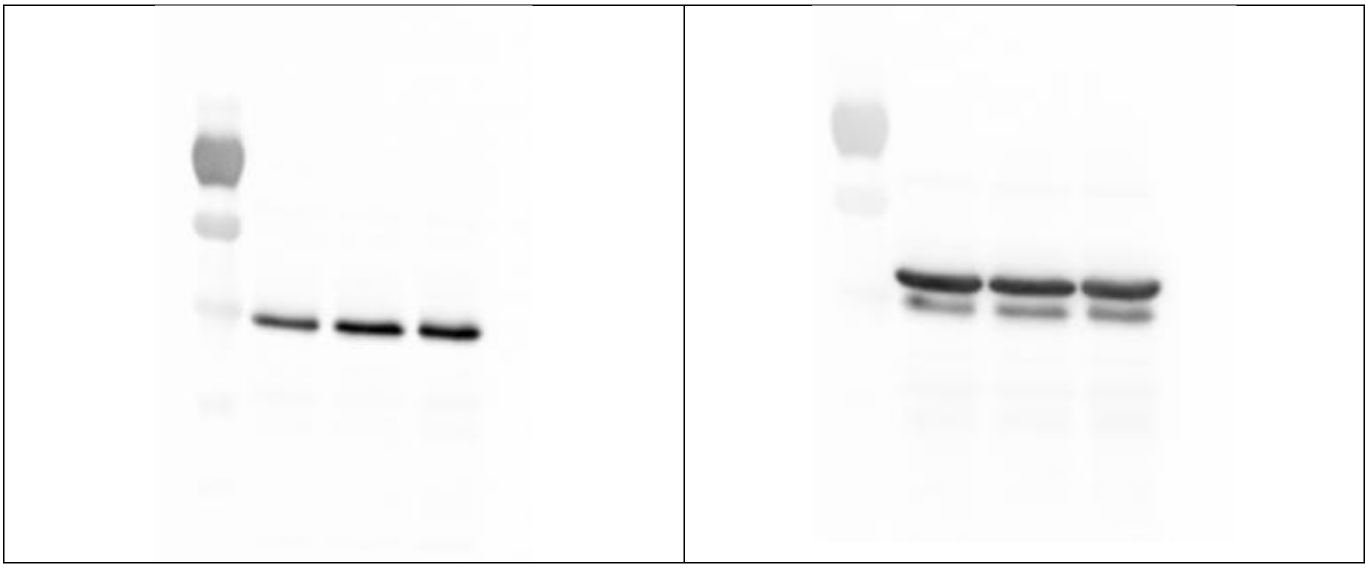

Supplementary figure 5a

pATM	ATM
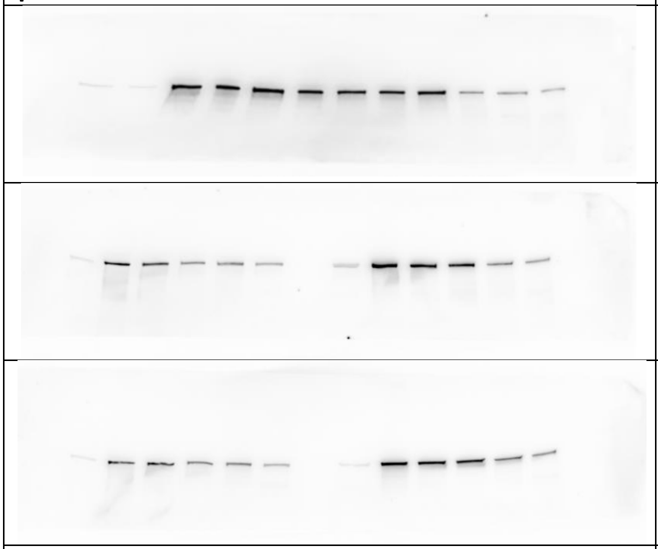	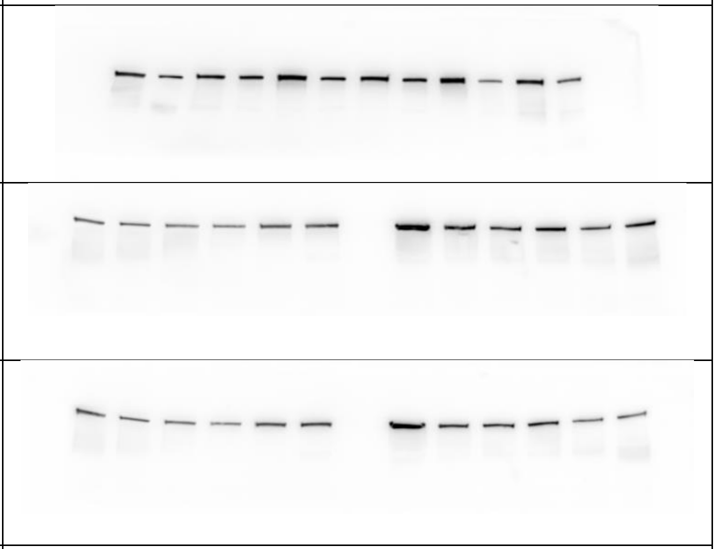
pChk2	Chk2
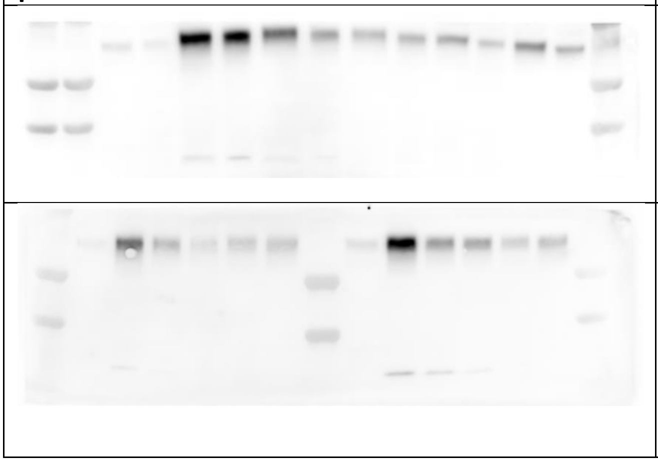	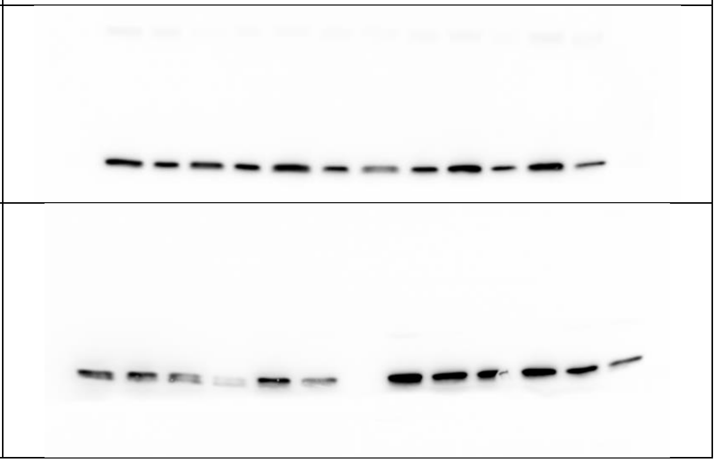

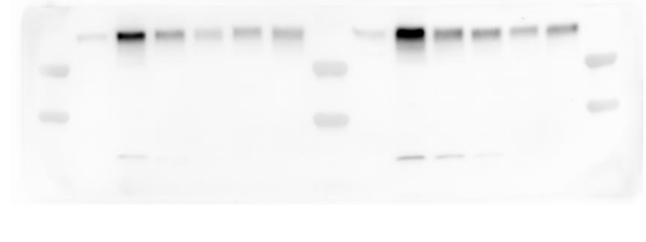

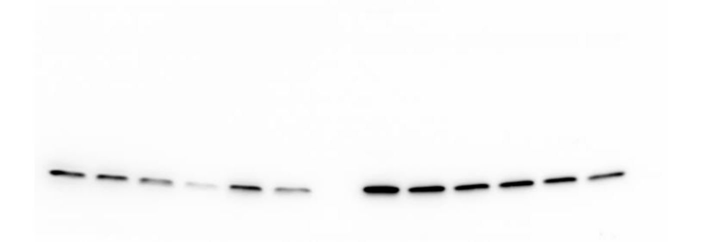

**pATR**	**ATR**

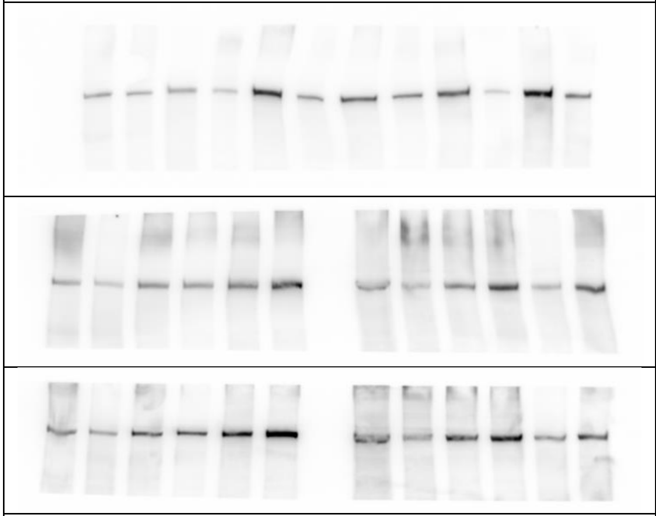

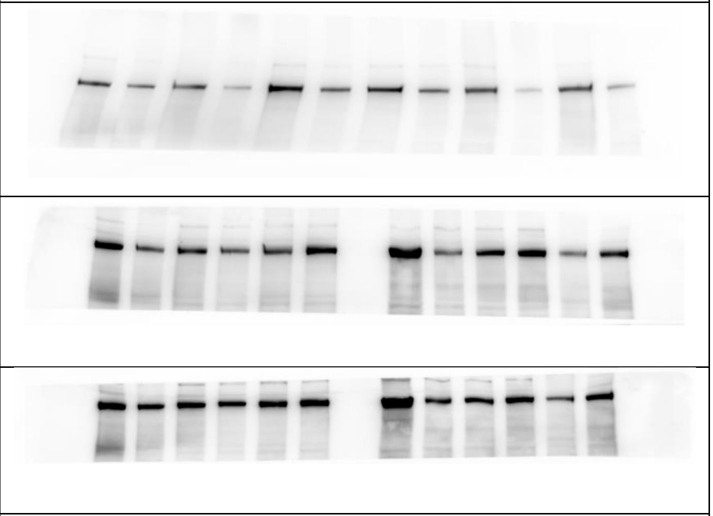

**pChk1**	**Chk1**

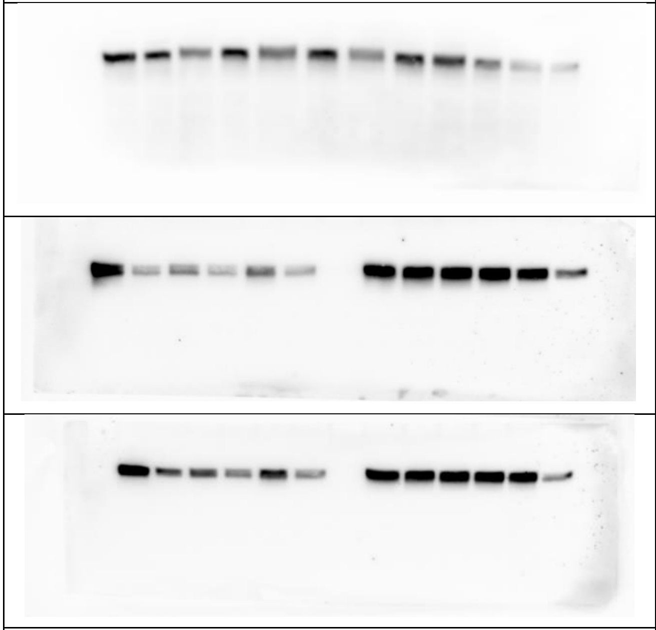

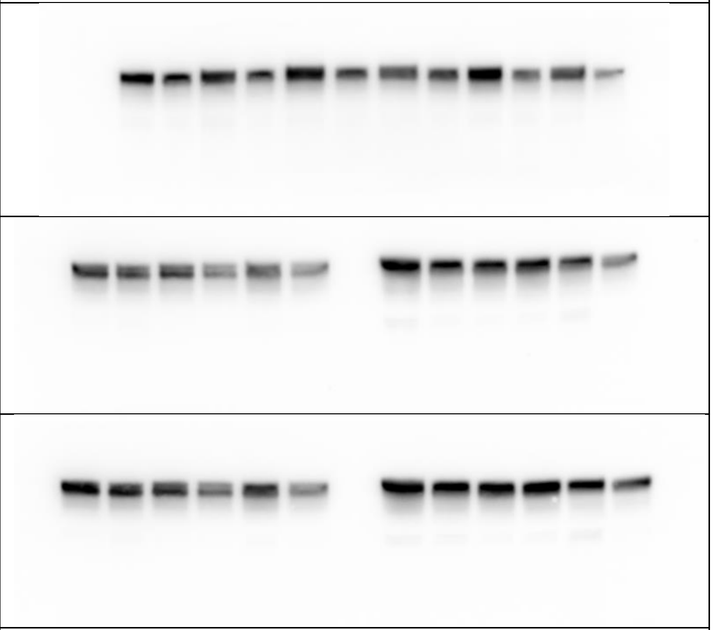

**Actin**	**Actin**

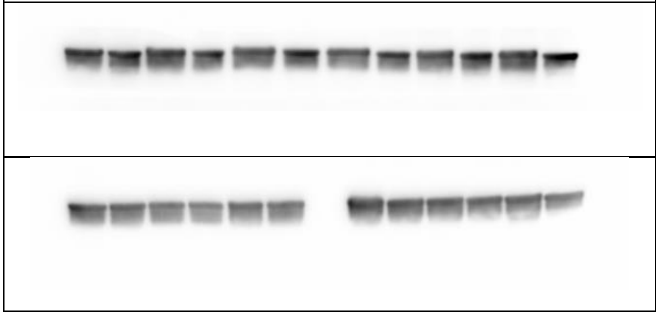

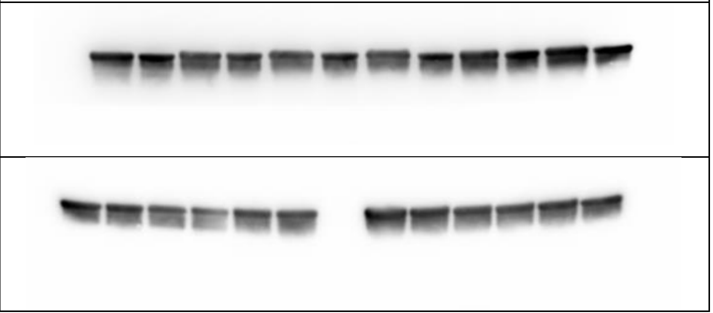

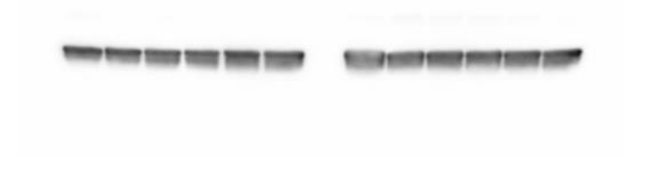	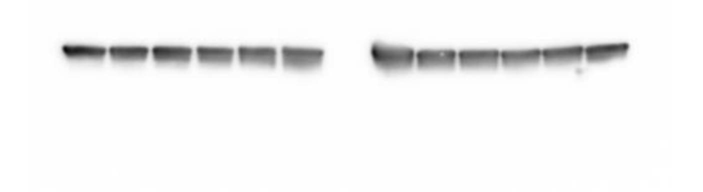

Supplementary figure 6a

p53	Actin
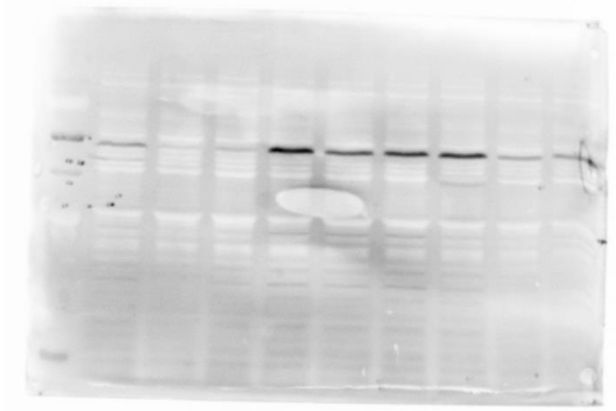	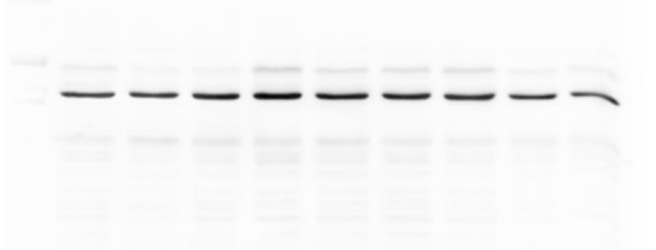
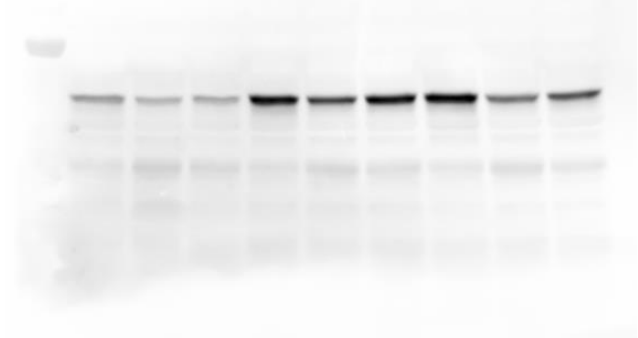	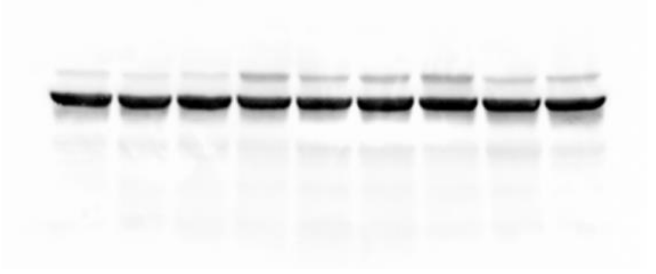
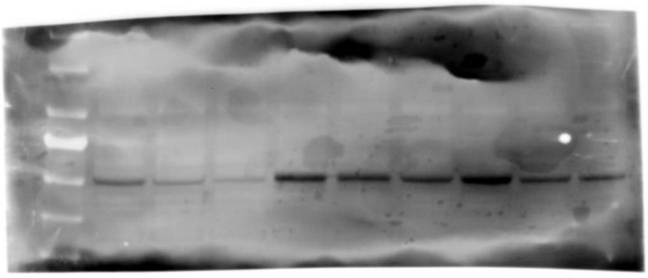	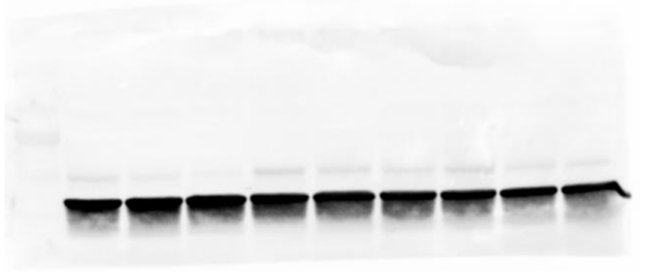
