# Supplementary material for: Syntaxin 18 regulates the DNA damage response and epithelial-to-mesenchymal transition to promote radiation resistance of lung cancer
Source: Cell Death Dis. 2022 Jun 6;13(6):529. doi: 10.1038/s41419-022-04978-4 (PMC9170725; doi:10.1038/s41419-022-04978-4)
Supplement: Supplementary file 7 — Dataset 1 [file 41419_2022_4978_MOESM7_ESM.docx]

**Raw data file**


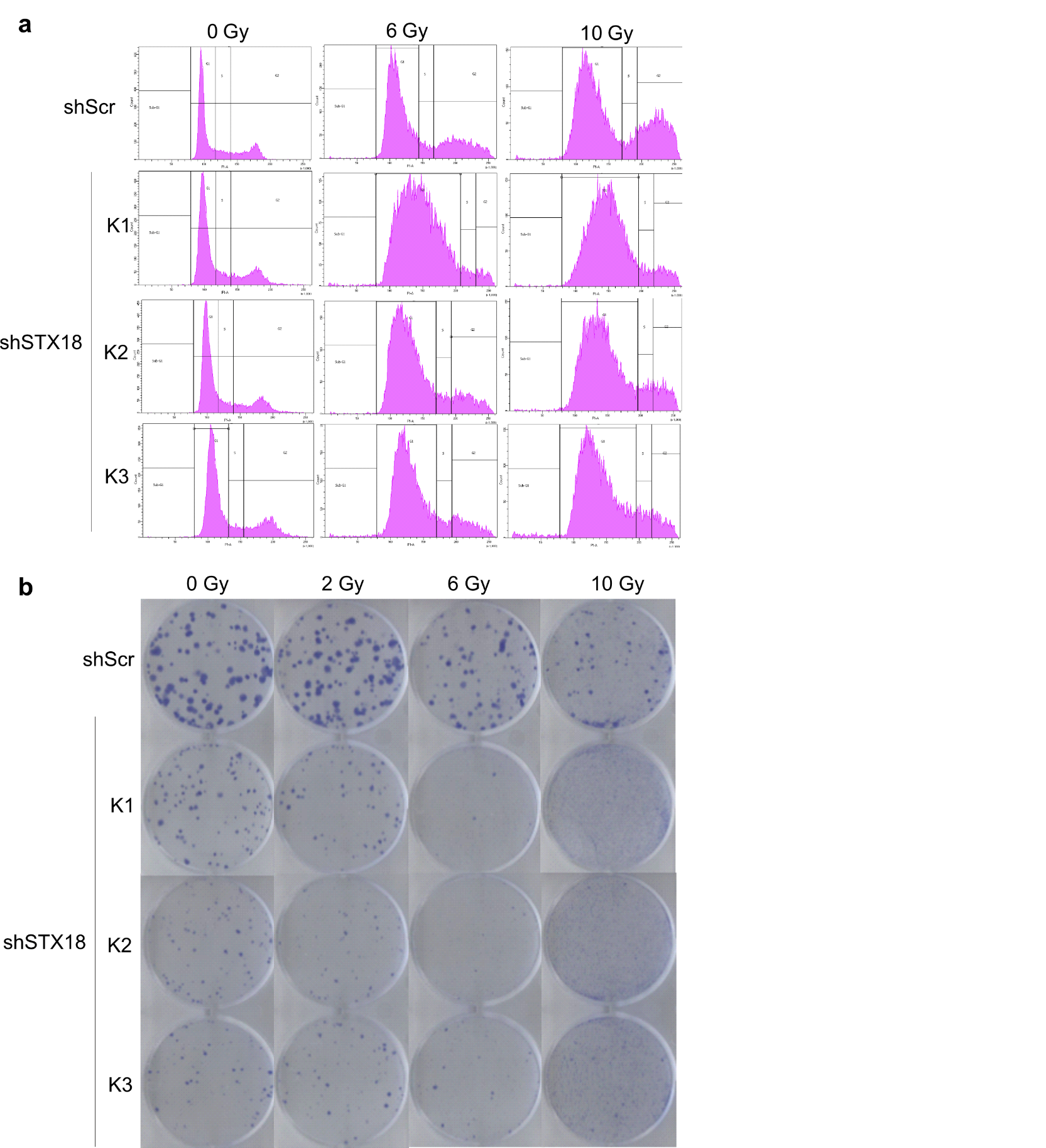


**Representative figure of Figure 2 c and d (A549).**

**a.** Representative pictures of A549 cell cycle analysis. **b.** Representative pictures of A549 colony assays.


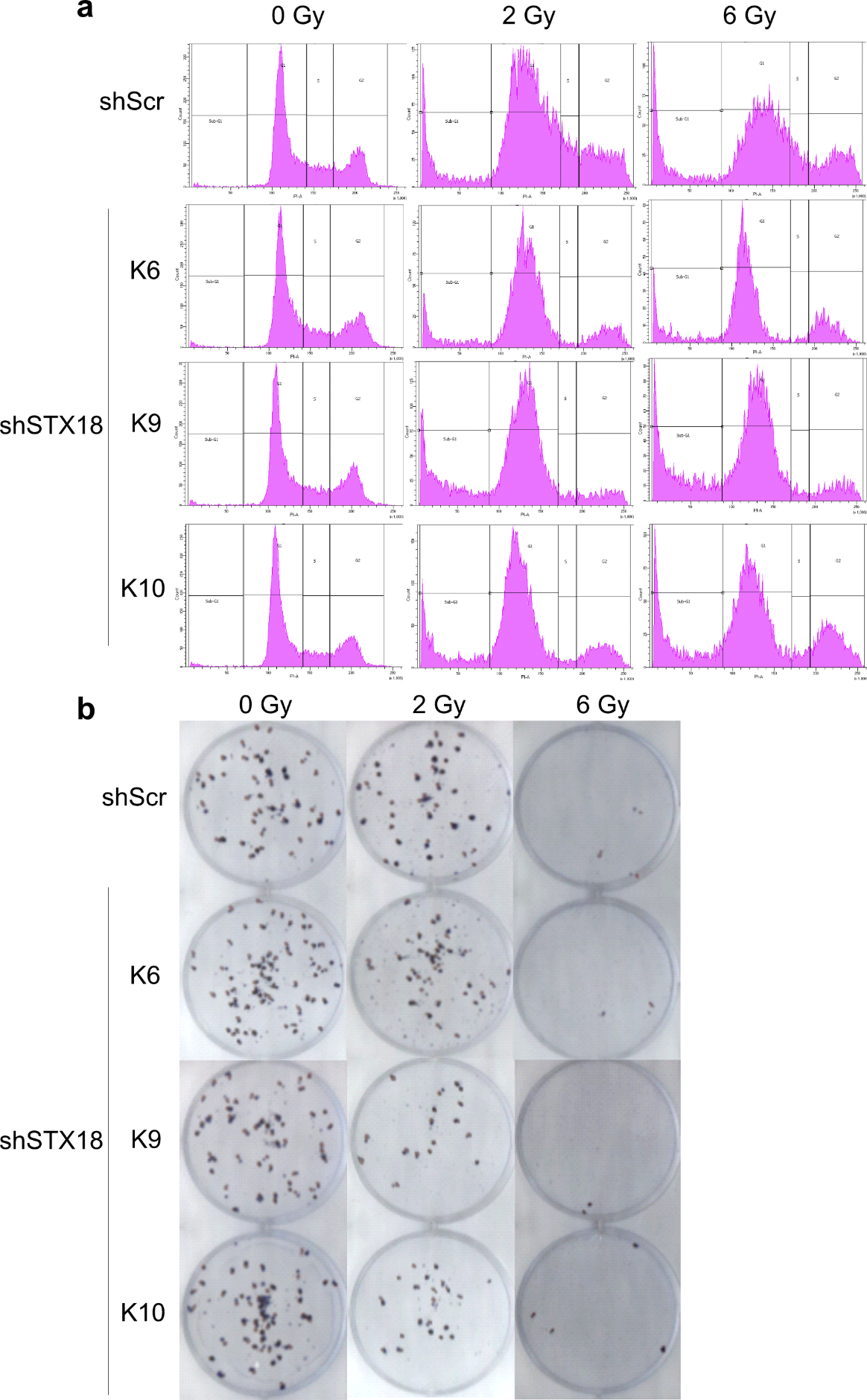


**Representative figure of Figure 2 c and d (H460).**

**a.** Representative pictures of H460 cell cycle analysis. **b.** Representative pictures of H460 colony assays.


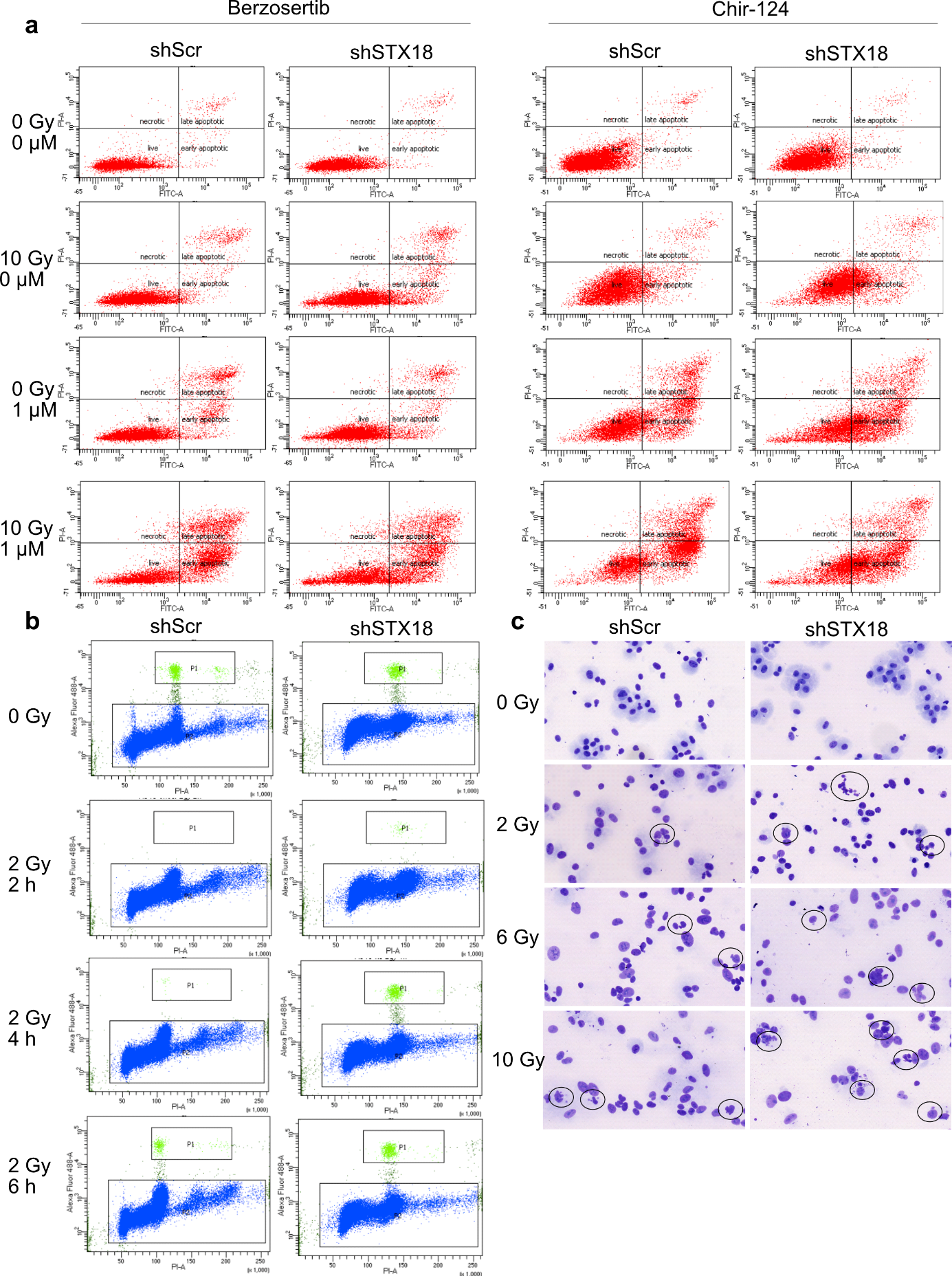


**Representative figure of Figure 4 b, c and d.**

**a.** Representative pictures of A549 Annexin V analysis. **b.** Representative pictures of H3PS10 staining. **c**. Representative pictures of fragmented nuclei quantification. Examples of fragmented nuclei are marked with a black circle.


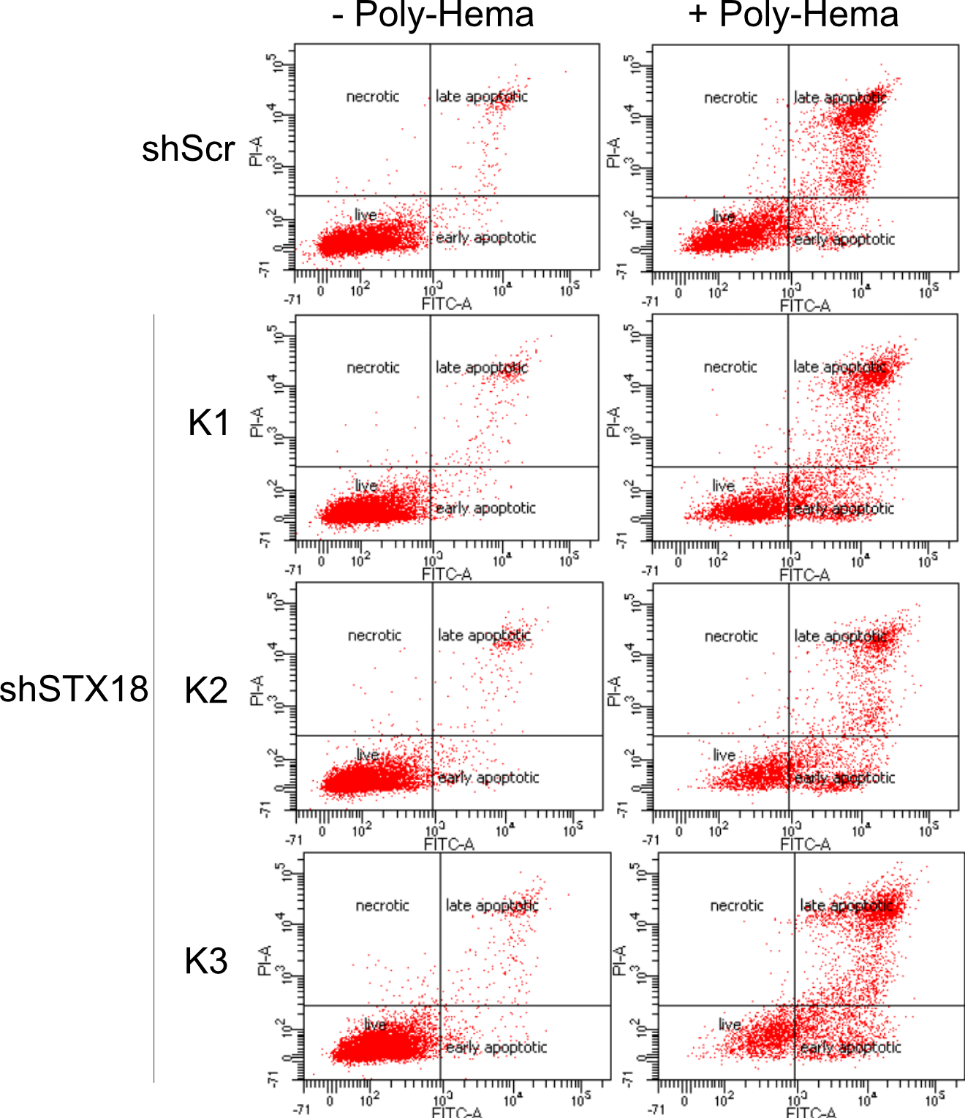


**Representative figure of Figure 5c.**

Representative pictures of Annexin V analysis.

**
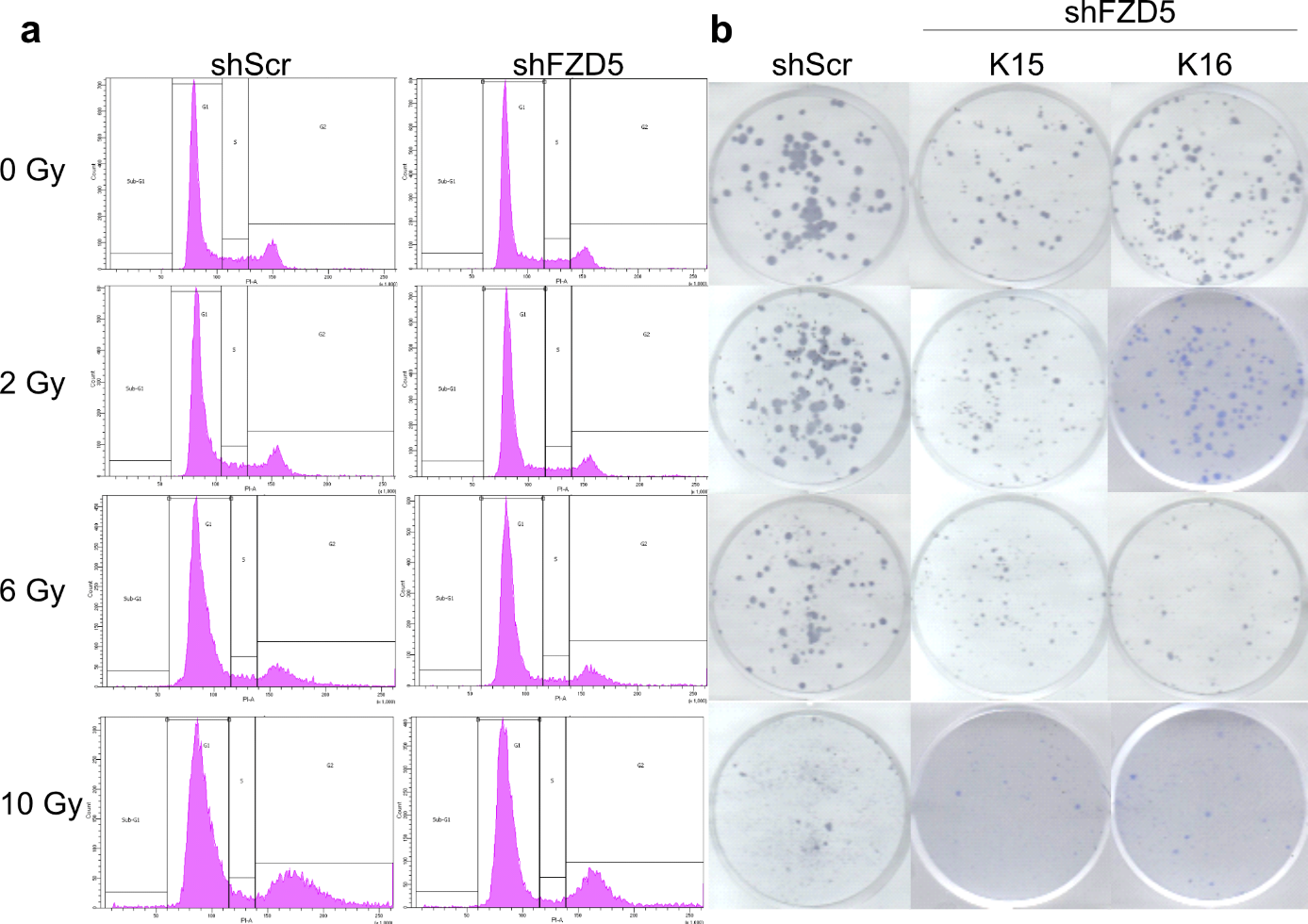
**

**Representative figure of Supplementary Figure 1 b and c. a.** Representative pictures of A549 cell cycle analysis. **b.** Representative pictures of A549 colony assays.


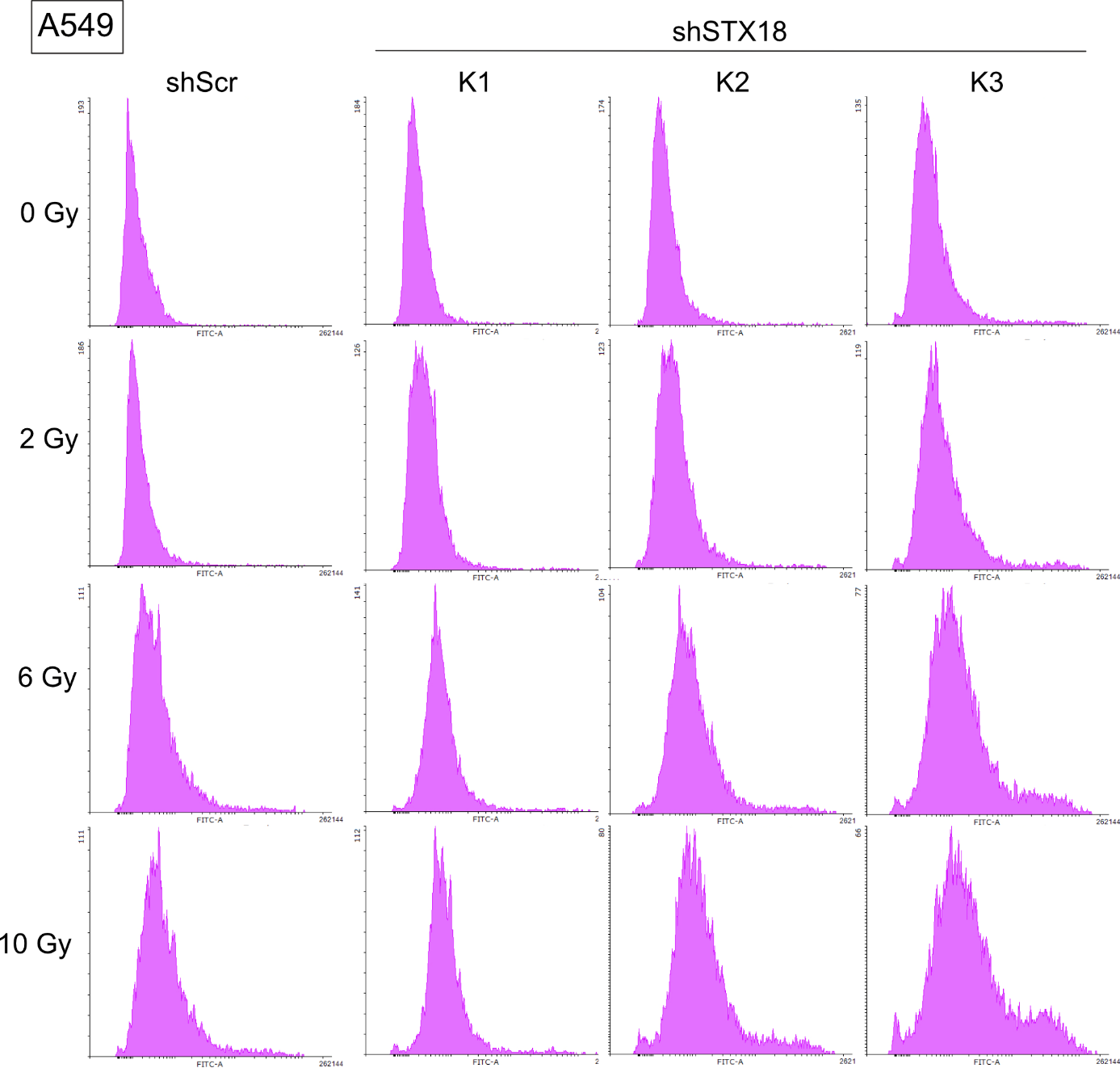


**Representative figure of Supplementary Figure 2 (A549).**

Representative pictures of A549 Annexin V analysis.


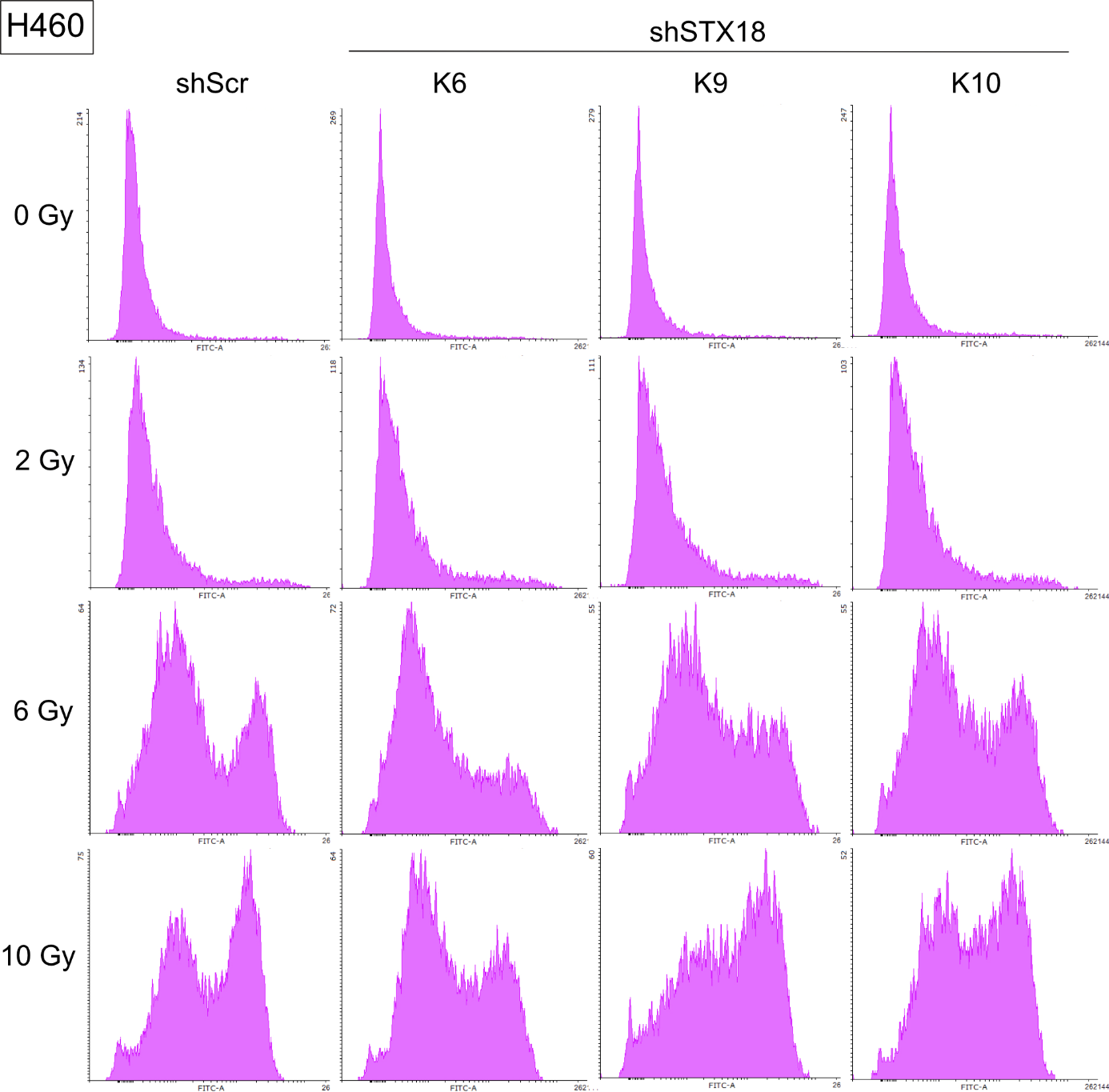


**Representative figure of Supplementary Figure 2 (H460).**

Representative pictures of H460 Annexin V analysis.


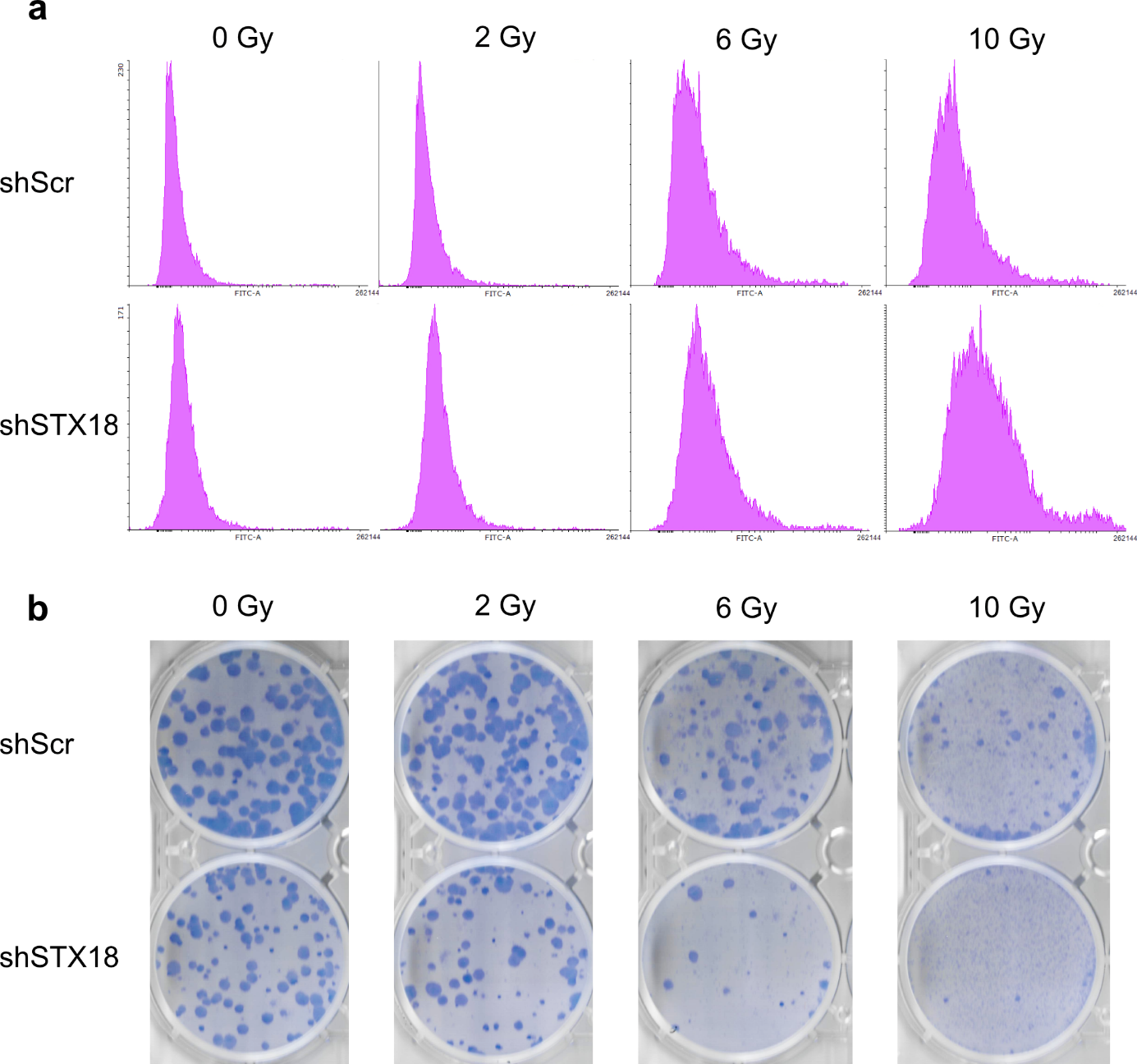


**Representative figure of Supplementary Figure 3 c and d.**

**a.** Representative pictures of A549 Annexin V analysis. **b.** Representative pictures of A549 colony assays.


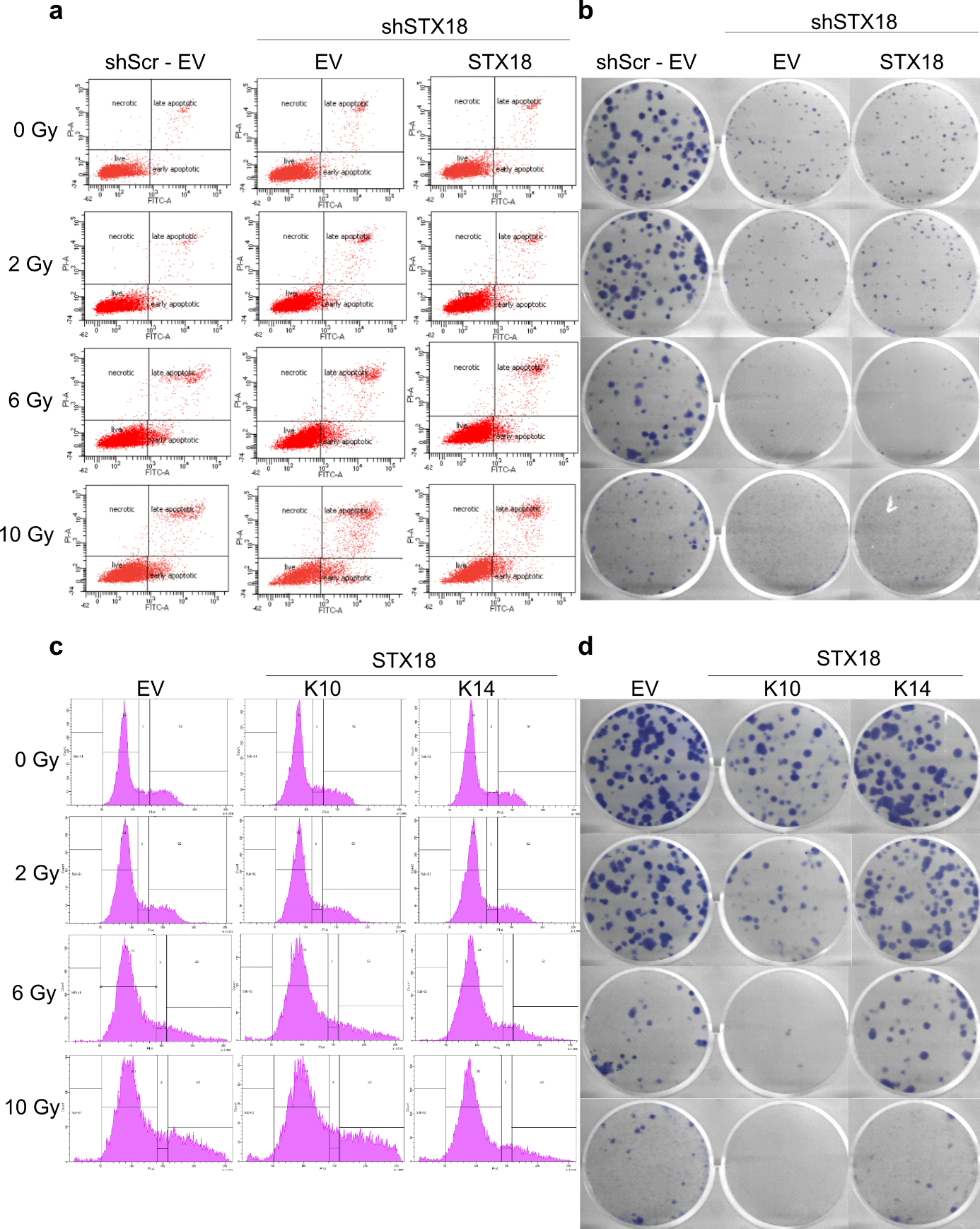


**Representative figure of Supplementary Figure 4 c, d (STX18 reintroduction), g and h (STX18 overexpression). a.** Representative pictures of A549 Annexin V analysis. **b.** Representative pictures of A549 colony assays. **c.** Representative pictures of A549 cell cycle analysis. **d.** Representative pictures of A549 colony assays.


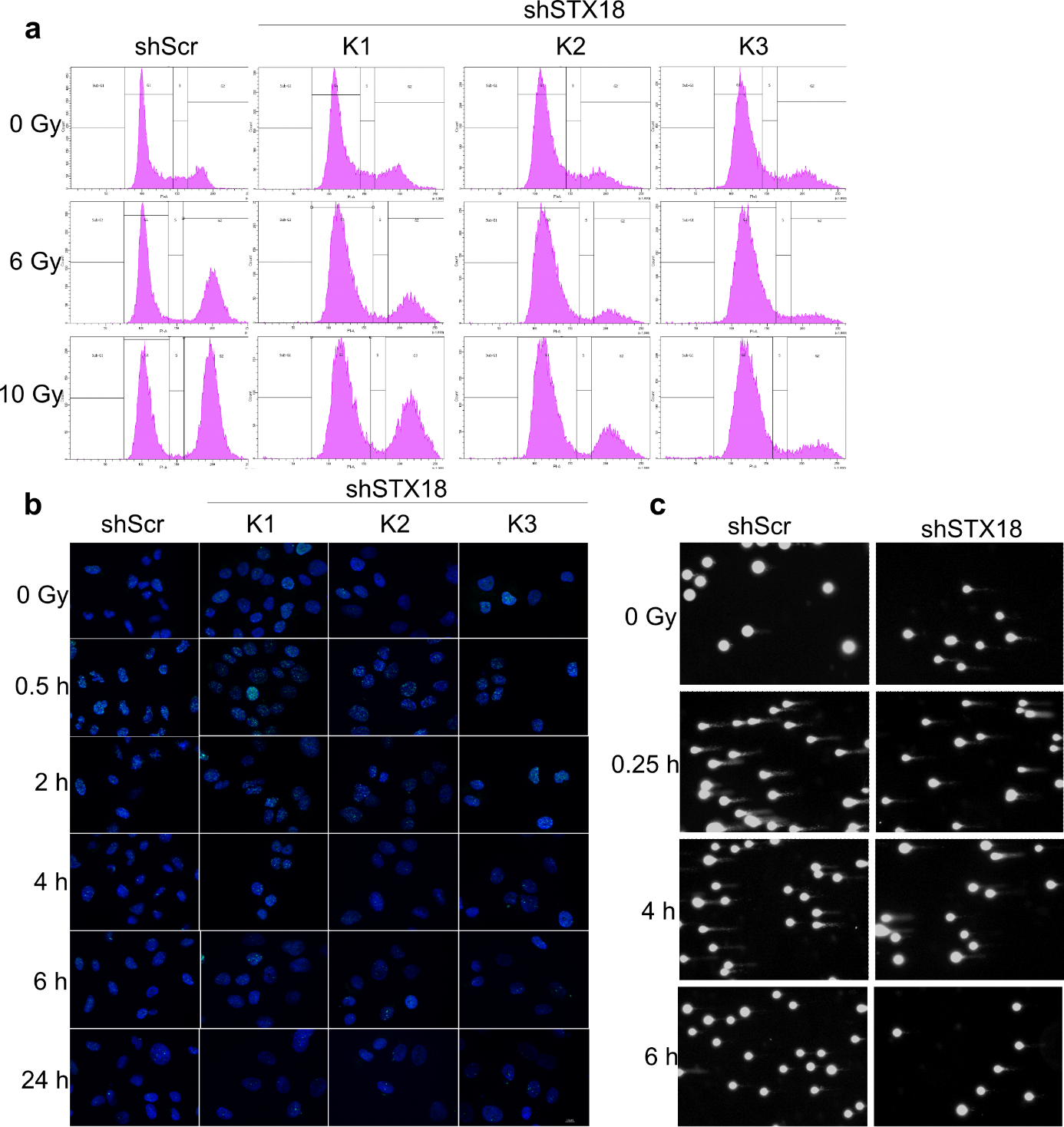


**Representative figure of Supplementary Figure 6 b, c/d and e/f.**

**a.** Representative pictures of A549 cell cycle analysis. **b.** Representative pictures of γH2A.X foci staining. **c.** Representative pictures of comet assays.
