# Supplementary material for: Syntaxin 18 regulates the DNA damage response and epithelial-to-mesenchymal transition to promote radiation resistance of lung cancer
Source: Cell Death Dis. 2022 Jun 6;13(6):529. doi: 10.1038/s41419-022-04978-4 (PMC9170725; doi:10.1038/s41419-022-04978-4)
Supplement: Supplementary file 9 — Author contributions [file 41419_2022_4978_MOESM9_ESM.docx]

**Author contributions**

C.T-H. designed, planned and performed the experiments, analyzed data, and wrote the paper. S.O. planned and performed the experiments, data analysis and was involved in interpretation. S.K. designed, planned and performed experiments. J.F. performed bioinformatics analysis. F.K. performed experiments. A.Sa. provided material and was involved in discussion. A.Sch. provided reagents, designed experiments, supported data interpretation, and wrote the paper. M.S. conceptualized and initiated the study, provided reagents, financial and administrative support, interpreted the data, and wrote the paper.
